# Comparison of Eight Technologies to Determine Genotype at the UGT1A1 (TA)_n_ Repeat Polymorphism: Potential Clinical Consequences of Genotyping Errors?

**DOI:** 10.3390/ijms21030896

**Published:** 2020-01-30

**Authors:** Tristan M. Sissung, Roberto H. Barbier, Douglas K. Price, Teri M. Plona, Kristen M. Pike, Stephanie D. Mellott, Ryan N. Baugher, Gordon R. Whiteley, Daniel R. Soppet, David Venzon, Arlene Berman, Arun Rajan, Giuseppe Giaccone, Paul Meltzer, William D. Figg

**Affiliations:** 1 Clinical Pharmacology Program, National Cancer Institute, National Institutes of Health, Bethesda, MD 20892, USA; sissungt@mail.nih.gov; 2 Molecular Pharmacology Section, National Cancer Institute, National Institutes of Health, Bethesda, MD 20892, USA; roberto.barbier@nih.gov (R.H.B.); dkprice@mail.nih.gov (D.K.P.); 3 CLIA Molecular Diagnostics Laboratory, Frederick National Laboratory for Cancer Research, Leidos Biomedical Research Inc., Frederick, MD 21702, USApikek@mail.nih.gov (K.M.P.); stephanie.mellott2@nih.gov (S.D.M.); ryan.baugher@nih.gov (R.N.B.); whiteleg@mail.nih.gov (G.R.W.); 4 Genomics Laboratory, Cancer Research Technology Program, Frederick National Laboratory for Cancer Research, Leidos Biomedical Research Inc., Frederick, MD 21702, USA; soppetdr@mail.nih.gov; 5 Biostatistics and Data Management, National Cancer Institute, National Institutes of Health, Rockville, MD 20850, USA; venzond@mail.nih.gov; 6 Office of Research Nursing in the Office of the Clinical Director, National Cancer Institutes, National Institutes of Health, Bethesda, MD 20892, USA; arleneb@mail.nih.gov; 7 Thoracic and Gastrointestinal Oncology Branch, National Cancer Institute, National Institutes of Health, Bethesda, MD 20892, USA; rajana@mail.nih.gov; 8 Department of Medicine, Weill Cornell Medicine, New York, NY 10065, USA; gig4001@med.cornell.edu; 9 Genetics Branch, National Cancer Institute, Bethesda, MD 20814, USA; pmeltzer@mail.nih.gov

**Keywords:** UGT1A1, pharmacogenomics

## Abstract

To ensure accuracy of *UGT1A1 (TA)_n_* (rs3064744) genotyping for use in pharmacogenomics-based irinotecan dosing, we tested the concordance of several commonly used genotyping technologies. Heuristic genotype groupings and principal component analysis demonstrated concordance for Illumina sequencing, fragment analysis, and fluorescent PCR. However, Illumina sequencing and fragment analysis returned a range of fragment sizes, likely arising due to PCR “slippage”. Direct sequencing was accurate, but this method led to ambiguous electrophoregrams, hampering interpretation of heterozygotes. Gel sizing, pyrosequencing, and array-based technologies were less concordant. Pharmacoscan genotyping was concordant, but it does not ascertain *(TA)_8_* genotypes that are common in African populations. Method-based genotyping differences were also observed in the publication record (*p* < 0.0046), although fragment analysis and direct sequencing were concordant (*p* = 0.11). Genotyping errors can have significant consequences in a clinical setting. At the present time, we recommend that all genotyping for this allele be conducted with fluorescent PCR (fPCR).

## 1. Introduction

The uridine diphosphate glucuronosyltransferase 1A1 (*UGT1A1*) gene is involved in glucuronidation of a wide variety of substances, including drugs, endobiotics, dietary, and environmental compounds [1]. Glucuronidation both inactivates [2] and facilitates clearance of UGT1A1 substrates [3]. Genetic variation in *UGT1A1* is associated with wide variability in UGT1A1 enzymatic activity that affects the pharmacokinetics and activity of over 50 therapeutics, including anticancer agents, antiretrovirals, NSAIDs, corticosteroids, anti-inflammatory agents, and many others [1]. Several agents now mention *UGT1A1* genotyping in their package insert and several hundred published studies are devoted to UGT1A1-related pharmacogenomics associations [1,4,5,6,7,8,9,10,11]. As we continue to make progress with implementation of clinical pharmacogenomics at the NIH Clinical Center, *UGT1A1* genotype accuracy is an important issue to address [12].

Variation in TA-repeat length of a critical TATA box in the *UGT1A1* promoter (rs3064744) significantly contributes to enzyme activity by altering the expression of *UGT1A1* and, thus, its enzyme activity. Individuals carrying (TA)_5_ or (TA)_6_ at this locus (UGT1A1*36 and UGT1A1*1, respectively; Table 1) have the greatest expression of *UGT1A1* compared to those carrying (TA)_7_ or (TA)_8_ (UGT1A1*28 and UGT1A1*37, respectively) [13,14]. Consequently, UGT1A1 enzymatic efficiency may be classified into three categories on the basis of rs3064744: (1) normal metabolism (NM) status for UGT1A1*36/*36, UGT1A1*36/*1, and UGT1A1*1/*1 carriers; (2) intermediate metabolism (IM) status for UGT1A1*36/*28, UGT1A1*36/*37, UGT1A1*1/*28, and UGT1A1*1/*37; (3) and poor metabolism (PM) status for UGT1A1*28/*28, UGT1A1*28/*37, and UGT1A1*37/*37 [15]. Other single nucleotide polymorphisms (e.g., UGT1A1*60 (3279T>G in phenobarbital response element; rs4124874), UGT1A1*93 (rs10929302), and UGT1A1*6 (Gly71Arg; rs4148323)) may also be involved in genetic determination of UGT1A1 glucuronidation capacity [1].

During assay development for two genotype-directed clinical trials involving a liposomal formulation of irinotecan (MM-398; NCT03221400) and an SN-38 conjugated HSP90 inhibitor (PEN-866; NCT02631733), we noted several instances in which different genotyping technologies reported different results for rs3064744 in the same control samples. We therefore undertook a process to determine which genotyping technologies were concordant: direct sequencing, pyrosequencing, gel sizing, DMET Plus arrays, Pharmacoscan arrays, Illumina (MiSeq), fragment analysis, and fluorescent PCR (fPCR).

## 2. Results

### 2.1. Genotyping Results

Genotyping data were obtained on *UGT1A1* rs3064744 (i.e., UGT1A1*36, UGT1A1*1, UGT1A1*28, and UGT1A1*37) from eight different genotyping platforms: direct sequencing, Illumina (MiSeq), fragment analysis, fluorescent PCR (fPCR; Quest Diagnostics), pyrosequencing, Agilent’s Bioanalyzer Lab-on-a-Chip Platform (analysis of Qiagen Pyromark PCR products in conjunction with Pyrosequencing data), DMET Plus (Thermo Fisher Scientific, Waltham, MA, USA), and Pharmacoscan (Thermo Fisher Scientific; Table 2). Choice of test was based on current methods used for screening of *UGT1A1* rs3064744 in clinical samples (Table S1). Genotypes were in Hardy–Weinberg equilibrium for all platforms when unique races were considered (*p* ≥ 0.14). Hardy–Weinberg testing was not possible for DMET Plus because specific *UGT1A1* genotypes are not reported (see Table 2 legend), as well as for genotyping platforms that were used to test certain genotype categories: fPCR and Pharmacoscan. Allele frequencies of UGT1A1*1 (i.e., (TA)_6_) obtained by Illumina sequencing matched previously-reported data (*p* > 0.178) for Caucasians (wild-type allele frequency (*1; (TA)_6_) = 0.76 vs. 0.70) [16], African Americans (wild-type allele frequency (*1; (TA)_6_) = 0.38 vs. 0.50) [17], Hispanics (wild-type allele frequency (*1; (TA)_6_) = 0.70 vs. 0.62) [18], and Asians (wild-type allele frequency (*1; (TA)_6_) = 0.78 vs. 0.87) [16]. 

### 2.2. Genotype Concordance between Illumina Sequencing, Fragment Analysis, and Fluorescent PCR

**Figure 1:**
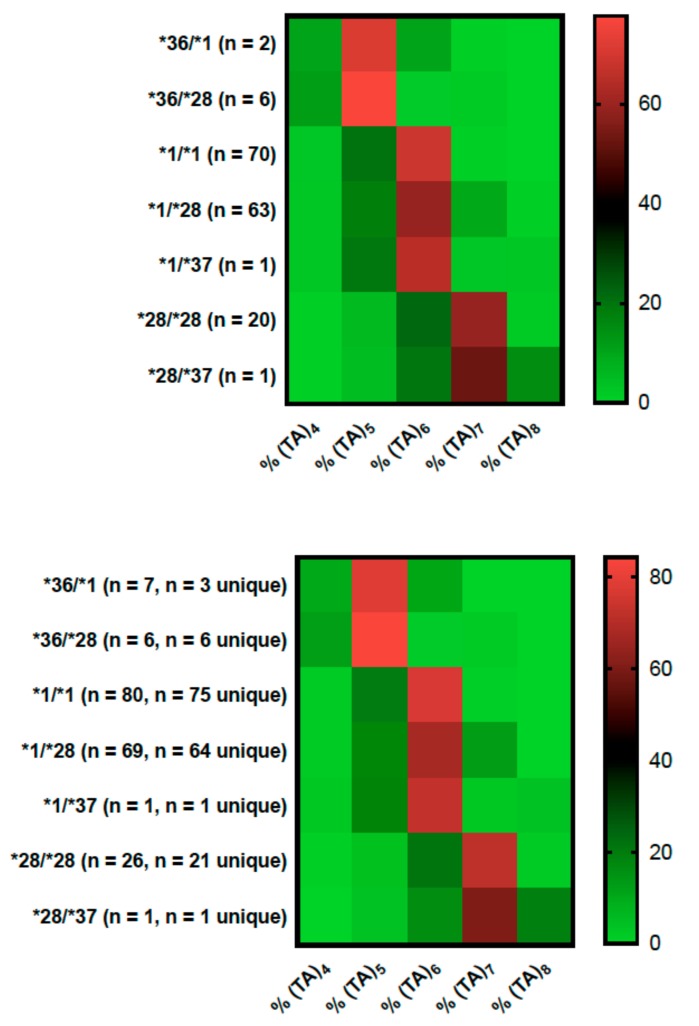
Heatmaps representing the % (TA)_n_ in (A) Illumina sequencing and (B) fragment analysis. Controls (for UGT1A1*36/*1, UGT1A1*1/*1, UGT1A1*1/*28, UGT1A1*28/*28) were repeated in multiple assays for fragment analysis; hence, there are differences in experimental replicates.

Shorter PCR fragments were preferentially amplified in both Illumina sequencing and fragment analysis (Figure 1). For example, Illumina sequencing of a known (TA)_6_ homozygote revealed a range of fragment sizes that are attributed to PCR “slippage”: 2.5% (TA)_4_, 18.1% (TA)_5_, 57.5% (TA)_6_, 11.7% (TA)_7_, and 0.3% (TA)_8_. However, beause distributions of fragment size percentages had non-overlapping ranges, we developed an algorithm that utilizes raw percentages of fragment sizes (Table S2) to assign genotypes to Illumina MiSeq data and fragment analysis. Fragment analysis was also conducted using commercially available controls (Table S3). Heuristic groupings were confirmed using principal component analysis, although principal components did not distinguish well between (TA)_5_/(TA)_6_ and (TA)_5_/(TA)_7_ genotypes in Illumina data due to the small number of carriers. (Figure S1).

The consistency between percentages of fragment sizes between the two genotyping methods were remarkable and suggest that the polymerase used in both assays was similarly error prone. Such analysis led to complete concordance between the two genotyping methods (Table 3). We sent samples representing each genotype to Quest Diagnostics for determination of genotype using fluorescent PCR (UGT1A1*1/*36 n = 2, UGT1A1*36/*28 n = 2, UGT1A1*1/*1 n = 2, UGT1A1*1/*28 n = 3, UGT1A1*1/*37 n = 1, UGT1A1*28/*28 n = 2, UGT1A1*28/*37 n = 1). These results were also completely concordant with Illumina and fragment analysis data. We concluded that these methods were accurate on the basis of three lines of evidence: (1) the former three genotyping methods were completely concordant, (2) genotypes were concordant with controls, and (3) Illumina and fragment analysis demonstrated similar percentages of fragment size distributions that were unique enough to bin into different genotype categories.

### 2.3. Genotype Concordance in Direct Sequencing, Gel Sizing, Pyrosequencing, DMET Plus, and Pharmacoscan

**Figure 2:**
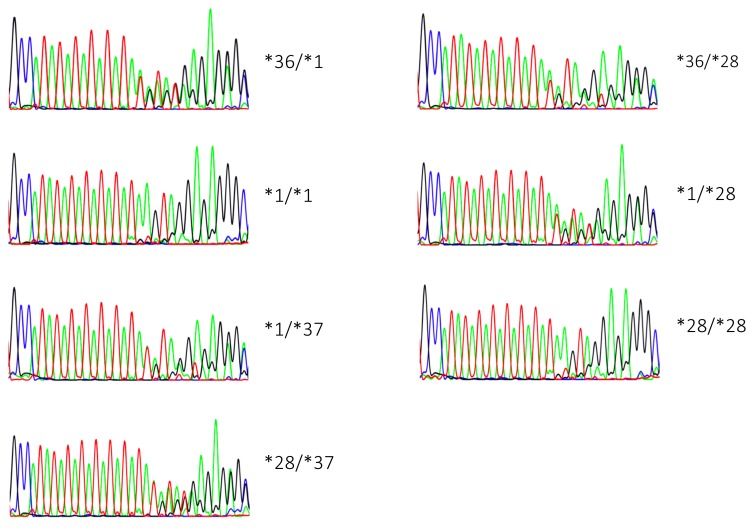
Representative electropherograms for each unique genotype.

As was previously observed [19], direct sequencing (in both directions) led to ambiguous genotyping in most heterozygotes as only nominal differences were apparent in electropherograms from heterozygotes with a common allele. Electropherograms from UGT1A1*1/*28 carriers were only nominally different from those of UGT1A1*1/*37 carriers, and the same was true for UGT1A1*36/*1 and UGT1A1*36/*28 carriers (Figure 2). In our hands, such minor differences resulted in significant genotyping uncertainty for rarely observed heterozygotes until sufficient genotype diversity allowed for comparison between a sufficient variety of genotypes. For several individuals who were homozygous on the basis of other sequencing methods (see Illumina and fragment analysis), genotypes were also ambiguous due to high background following the (TA)_n_ repeat. We attribute both problems to artifacts generated during PCR amplification.

**Figure 3:**
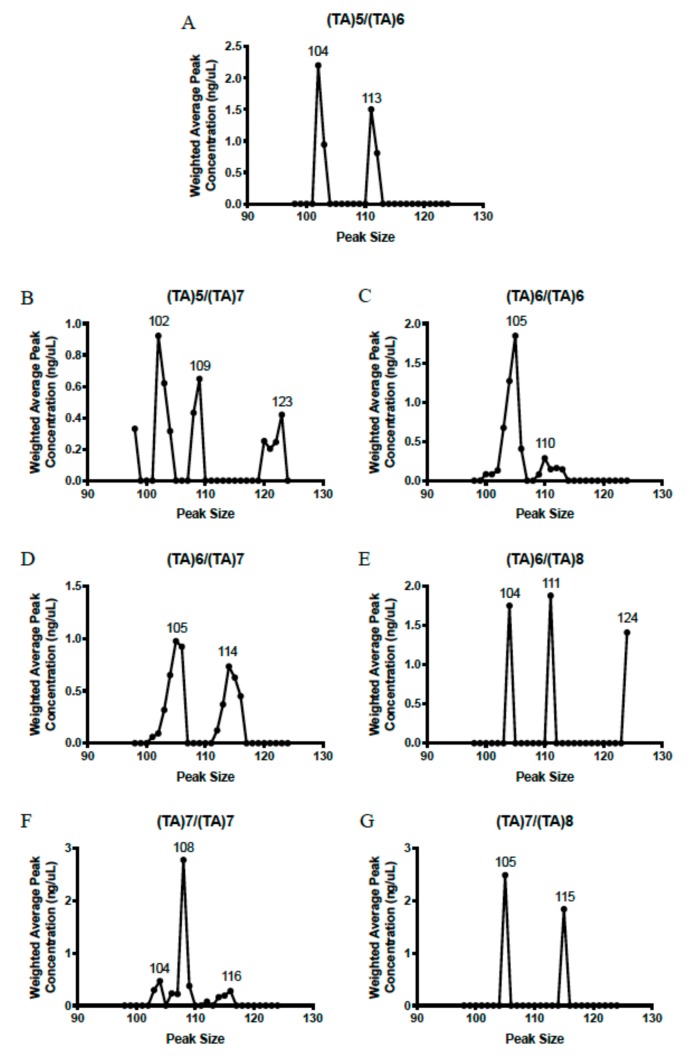
Weighted averages of each peak size were calculated for all samples that underwent gel sizing analysis. Genotypes represented in this figure were determined by Illumina (MiSeq), fragment analysis, and fPCR, as gel sizing was ineffective on its own: (**A**) UGT1A1*36/*1, (**B**) UGT1A1*36/*28, (**C**) UGT1A1*1/*1, (**D**) UGT1A1*1/*28, (**E**) UGT1A1*1/*37, (**F**) UGT1A1*28/*28, (**G**) UGT1A1*28/*37.

Bioanalyzer gel sizing of Pyromark samples (examples of each genotype in Figure S2) demonstrated wide variability in fragment sizing and highly inconsistent peak sizing (Figure 3). For instance, UGT1A1*36/*1 (Figure 3A) demonstrated two band sizes (104–105 and 113–114), but the UGT1A1*36/*28 genotype had two lower-sized peaks (102-104 and 108-109) along with a larger band at 120-123 base pairs (Figure 3B). Yet, other UGT1A1*28 carriers did not have bands between 120-123 base pairs (Figure 3B,F,G). Many other inconsistencies are clear from the data and precluded calling genotypes.

**Figure 4:**
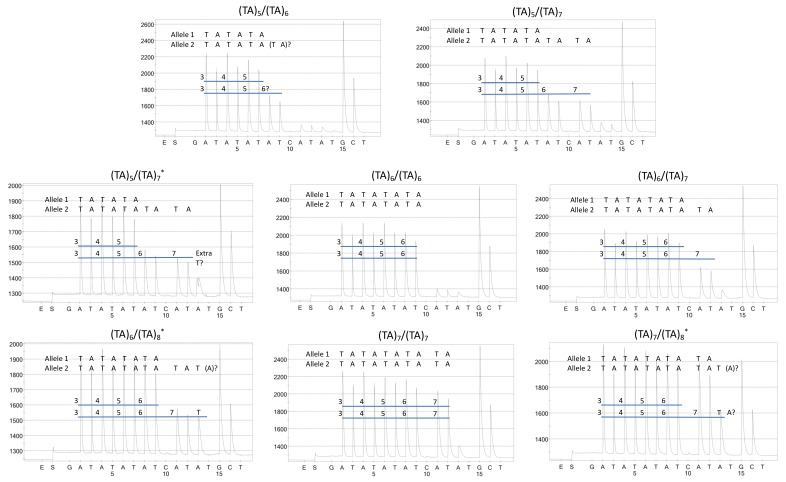
Pyrograms from representative genotypes (as called by MiSeq, fragment analysis, and/or fPCR): (**A**) UGT1A1*36/*1, (**B**) UGT1A1*36/*28, (**C**) UGT1A1*36/*28 (ambiguous), (**D**) UGT1A1*1/*1, (**E**) UGT1A1*1/*28, (**F**) UGT1A1*1/*37, (**G**) UGT1A1*28/*28, (**H**) UGT1A1*28/*37. * The current UGT1A1*36/*28 sample was uncalled by pyrosequencing due to the extra T peak, but five other samples were appropriately called; the single UGT1A1*1/*37 sample (shown) was uncalled by pyrosequencing due to the lack of an “A” peak; the single UGT1A1*28/*37 sample (shown) was uncalled by pyrosequencing due to the lack of an “A” peak.

Five of six individuals harboring UGT1A1*36/*28 were called using pyrosequencing (Figure 4B); however, an individual who carried UGT1A1*36/*28 had an extra T peak on the pyrogram that resulted in an ambiguous call (Figure 4C). A lack of an extra “A” peak was observed in (TA)_6_/(TA)_8_, whereas an extra T peak was observed in a (TA)_7_/(TA)_8_ carrier (Figure 4F,G, respectively). We were able to call *n* = 155 genotypes using pyrograms, which were completely concordant with Illumina sequencing and fragment analysis, but *n* = 7 genotypes were not called due to ambiguity in fragment sizes (*n* = 1 UGT1A1*36/*28, *n* = 1 UGT1A1*1/*1, *n* = 1 UGT1A1*1/*37, *n* = 2 UGT1A1*1/*28, *n* = 1 UGT1A1*28/*28, and *n* = 1 UGT1A1*28/*37; Table 3). Thus, pyrosequencing is accurate, but it is often ambiguous for (TA)_7_ and (TA)_8_ fragment sizes.

The DMET Plus array does not provide specific genotype calls for the platform reports (TA)_5_ or (TA)_6_ and (TA)_7_ or (TA)_8_, which are useful in the determination of metabolism status (i.e., NM, IM, and PM). When metabolism status is considered alone, the DMET Plus was 94.2% concordant with Illumina and fragment analysis (Table 3), and all non-concordance was due to miscalls of UGT1A1*1/*28 (*n* = 4) and UGT1A1*36/*28 (*n* = 5), for which DMET Plus determined to be normal metabolizers (Table 3). These results are similar to a prior study that evaluated several single nucleotide polymorphisms using the DMET array (albeit version 1 and not DMET Plus) and direct sequencing [20]. We sent representative samples for genotyping (UGT1A1*36/*1 n = 1, UGT1A1*36/*28 n = 5, UGT1A1*1/*1 n = 3, UGT1A1*1/*28 n = 7, UGT1A1*1/*37 n = 1, UGT1A1*28/*28 n = 3, UGT1A1*28/*37 n = 1). Pharmacoscan was inconsistent with Illumina sequencing and fragment analysis in two patients carrying UGT1A1*1/*37 and UGT1A1*28/*37 genotypes as UGT1A1*1/*1 and UGT1A1*28/*28, respectively. This result was expected, as the latest version of the Affymetrix platform, Pharmacoscan, detects (TA)_5_, (TA)_6_, and (TA)_7_ individually, but it does not probe for (TA)_8_ repeats. We next engaged Nanostring (Nanostring Technologies, Seattle, USA) to design an assay detecting the *UGT1A1* rs3064744 allele. At that point, Nanostring did not support customized SNV assay development without submission of several thousand samples. 

### 2.4. Phenotype Concordance

In spite of the non-concordance between genotype, all assays were phenotypically concordant with two exceptions: (1) the DMET Plus array misclassified nine IMs (UGT1A1*1/*28 and UGT1A1*36/*28) as EMs, and (2) the Pharmacosan array misclassified an IM (UGT1A1*1/*37) as an EM. As the pyrosequencing assay often led to unclear results, UGT1A1 phenotype would be uncalled in seven cases using this technology. Direct sequencing and Pyromark gels would be likely to result in incorrect phenotypes in a multitude of cases. No platform for which we received raw data provided clear genotypes without significant post-processing, which could impede phenotype assessment in many cases.

### 2.5. Comparisons with Previously Published Peer-Reviewed Data

We next searched the literature for publications ascertaining *UGT1A1* rs3064744 in healthy American and European Caucasians (*n* = 11,145) and healthy individuals with African descent (*n* = 1707). We identified 136 publications appearing between 1996 and 2018 that genotyped this allele in populations who did not have disease or conditions directly related to UGT1A1 function (e.g., hyperbilirubinemia). A total of 71 of these publications contained data comparable with the present study and prior publications utilizing direct sequencing (26 studies), fragment analysis (52 studies), gel sizing (17 studies), or pyrosequencing (14 studies; Table S4). The genotype and allele frequencies differed substantially between the different methods applied to genotyping Caucasians (*P* < 0.0001). This difference was primarily attributable to gel sizing, which has a lower frequency of (TA)_6_/(TA)_6_ genotypes (43.0%) and a higher frequency of (TA)_6_/(TA)_7_ (47.7%) than others (%(TA)_6_/(TA)_6_ = 44.3, 43.8, 46.1; and %(TA)_6_/(TA)_7_ = 44.3, 43.9, 45.9 for direct sequencing, fragment analysis, and pyrosequencing, respectively). When gel sizing was excluded, the three others also differed (*P* = 0.0046). This difference was attributable to pyrosequencing, which has a higher frequency of (TA)_6_/(TA)_7_ and a lower frequency of (TA)_7_/(TA)_7_ than others (%(TA)_7_/(TA)_7_ = 11.5, 12.3, 9.3, and 8.0 direct sequencing, fragment analysis, gel sizing, and pyrosequencing, respectively). Data obtained from direct sequencing and fragment analysis did not differ in either Caucasians (*P* = 0.11) or individuals with African descent (*P* = 0.92). 

## 3. Discussion

The present study was undertaken to address concerns about genotype accuracy in two genotype-directed prospective clinical trials utilizing a liposomal irinotecan formulation (MM-398) and an HSP90 inhibitor conjugated to SN-38 (PEN-866; NCT03221400 and NCT02631733, respectively), for which we chose to use fPCR by Quest Diagnostics. Although we demonstrated that fPCR (Table S1) is an accurate genotyping method, our findings suggest that patients who undergo *UGT1A1* genotyping for dosing or therapeutic choice may be underserved by many other current genotyping technologies, regardless of the CLIA certification of the Illumina sequencing, fragment analysis, DMET analysis, and pyrosequencing used herein. Genotyping errors have the potential to persist in the medical record, as the germline does not change throughout an individual’s lifetime. Such errors can expose patients to a variety of iatrogenic hazards [15,21]. Thus, as pharmacogenomic research continues to progress and new *UGT1A1*-related gene–drug interactions are identified and characterized, selection of the appropriate genotyping test for *UGT1A1* rs3064744 is critical [1].

We showed that only fPCR provides unambiguous results that require no post-processing of data; albeit, the Nichols Institute, which conducted the test, does not disclose methods for this assay [22]. Although Illumina and fragment analysis were shown to be accurate, our results suggest that even these technologies can be easily misinterpreted due to amplification of multiple smaller fragment sizes that are not representative of a patient’s genotype. The present results also demonstrate that several technologies are inappropriate for use in obtaining specific genotypes at rs3064744: pyrosequencing, Pyromark gels, DMET Plus, and Pharmacoscan. Pyrosequencing provides many ambiguous calls due to the presence of additional peaks that convolute interpretation of pyrograms. Pyromark gel and bioanalyzer genotyping frequently demonstrate peaks that could fall into several genotype categories, and we could not confidently call genotype using this technology. Analysis of previously published studies also demonstrates that pyrosequencing and gel sizing results differ from those of other methods. Use of the DMET Plus array resulted in nine miscalls that all suggested IMs were EMs. Although Pharmacoscan has improved the specificity and accuracy of genotyping, it does not detect UGT1A1*37 alleles, and a patient who was UGT1A1*1/*37 (an IM) was called UGT1A1*1/*1 (an NM). Thus, even if one ignores specific genotypes and only classifies patients as NM, IM, or PM, then DMET Plus and Pharmacoscan could each introduce genotyping errors into the medical record. Because African Americans carry a higher frequency of (TA)_5_ and (TA)_8_ [23], this population is at particular risk of genotyping miscalls. As racial admixture becomes more prevalent in the United States [24], such genotyping errors are of particular concern.

Our results also extend to other clinical facets, including dosing and therapeutic selection of traditional irinotecan formulations. The Dutch Pharmacogenetics Working Group (DPWG) recommendations advise a 70% starting dose reduction for UGT1A1*28/*28 carriers receiving irinotecan, and further dosing is based on neutrophil count [25]. The French National Network of Pharmacogenetics (RNPGx) advises a 25%–30% dose reduction in UGT1A1*28/*28 carrier receiving 180–230 mg/m^2^ spaced by 2-3-week intervals and administering UGT1A1*1/*28 or UGT1A1*28/*28 carriers less than 240 mg/m^2^ [26]. Recommendations have not been established for other genotypes because data in UGT1A1*36 and UGT1A1*37 carriers is sparse. As doses are increased on the basis of neutrophil count, risk is focused in patients who experience excessive toxicity due to a high starting dose. Thus, genotyping errors are of particular concern for UGT1A1*28/*28 carriers receiving 180–230 mg/m^2^ and UGT1A1*1/*28 or UGT1A1*28/*28 carriers receiving more than 240 mg/m^2^ of irinotecan. DMET genotyping would have incorrectly reported that nine intermediate metabolizers were extensive metabolizers; thus, these patients could have been subjected to irinotecan doses greater than 240 mg/m^2^ that could have been dangerous. Other genotyping platforms, except fPCR, could have led to non-calls and/or incorrect calls, as these platforms are often ambiguous or incorrect. 

Atazanavir prescribing is also based on UGT1A1 metabolism status: NM, IM, and PM. Patients with PM status are at risk of developing jaundice that will result in atazanavir discontinuation (approximately 20%–60% of carriers), and alternate agents should be considered [15]. One patient in our cohort was called *1/*1 (NM) by Pharmacoscan, but was actually *1/*37 (IM) by other genotyping methods and ambiguous via pyrosequencing. Such a patient would not necessarily be at risk under the current dosing guidelines, but a patient carrying *37/*37 (PM) most likely would have been called *1/*1 and would have been treated improperly. Nevertheless, should a patient be assigned an incorrect genotype prior to atazanavir therapy, such an error would propagate in the medical record, potentially exposing such a patient to improper therapy in the future. 

As the rs3064744 locus appears to have significant phenotypic consequences on a wide variety of agents, genotyping errors at this site are of particular concern as pharmacogenomics testing continues to discover new interactions with this variant. At best, ambiguity in genotype testing would lead to unnecessary delays in therapy when urgent treatment is required [12]. At worst, incorrect genotypes could harm patients and be propagated in the medical record, potentially resulting in greater complications. Finally, the present lack of data on irinotecan outcome in UGT1A1*36 and UGT1A1*37 carriers is also likely a function of genotyping errors in the literature. At the present time, we recommend that all genotyping for this allele be conducted with fPCR. Technological innovation to overcome genotype miscalls at this site is urgently needed for both clinical and scientific purposes.

## 4. Materials and Methods

### 4.1. Patients and Samples

The patient cohort was derived from a prospective pharmacogenomics trial [27]. Briefly, 546 patients with histological diagnosis of primary lung carcinoma were enrolled between 2009 and 2012 (NCT#00923884). The study included individuals with a histological diagnosis of non-small cell (stage I-IV) or small cell lung cancer (limited or extensive stage) who received any treatment (surgical resection, chemotherapy, radiation, or molecularly targeted therapy), had any ECOG score (0–3), and had normal or impaired organ function. Patients were not precluded from enrolling if they had a history of diagnosis with other cancers. Of these, 163 patients who received paclitaxel were genotyped for a prior publication, and no associations between UGT1A1 genotypes and clinical outcomes or patient or disease parameters were detected [27]. Because the present study was concerned with genotyping methods, clinical outcomes were not considered relevant. The study was approved by the Institutional Review Board at the National Cancer Institute (Bethesda) and Veteran’s Affairs Medical Center (Washington D.C.) (protocol 09C0103, approved 5 March 2009), and all patients provided informed consent. Genomic DNA was extracted from blood samples using the QIAamp DNA Blood kit (Qiagen, Germantown, MD, USA). CLIA-certified fPCR was conducted by the Nichols Institute (Quest Diagnostics Inc, San Juan Capistrano, CA, USA), and Pharmacoscan was conducted by RUCDR Infinite Biologics (Nelson Biological Laboratories, Piscataway, NJ, USA).

### 4.2. Illumina Sequencing (MiSeq) 

Primers were designed for the region of interest within the promoter of UGT1A1, specifically dbSNP ID: rs3064744. For the MiSeq amplicon design, the FW primer sequence was 5′- TTTATCTCTGAAAGTGAACTC-3′ and the RV was 5′- TGGGCGTCCGCCCTGGGACTC-3′. These primers were adapted with M13FW and M13RV tags: 5′-gtaaaacgacggccagt-3′ (FW strand) and 5′-ggaaacagctatgaccatg-3′ (RV strand). All primers were purchased from Thermo Scientific. For the PCR, Invitrogen’s High-Fidelity Taq System (Thermo Fisher Scientific, Waltham, MA, USA) and 10 mM dNTPs (Thermo Fisher Scientific) with 5% molecular grade dimethyl sulfoxide (Sigma-Aldrich, St. Louis, MO, USA) were utilized. The M13-labeled primers were used to generate the target amplicons for the libraries from DNA with an Applied Biosystems Veriti 96-well thermal cycler (Thermo Fisher Scientific) using the following conditions: 95 °C for 5 min; followed by 20 cycles of 94 °C for 1 min, 58 °C for 1 min, 72 °C for 1 min; followed by 20 more cycles of 94 °C for 1 min, 65 °C for 1 min, 72 °C for 1 min, and completed with a final extension of 72 °C for 10 min, then holding at 4 °C. The resulting target specific amplicons were used directly in the adapter PCR that follows. 

For this step, barcoded primers for bi-directional coverage of each target amplicon were designed per Illumina indexing protocols (www.Illumina.com) using Illumina P5 and P7 adapter-indexes, barcodes, and M13 adapters. These adapters were added to the amplicons to create the library samples with the Veriti thermal cycler and the following conditions: 95 °C for 2 min; followed by 15 cycles of 94 °C for 30 s, 55 °C for 30 s, 72 °C for 1 min, and completed with a final extension of 72 °C for 1 min, then holding at 4 °C. The PCR system was Invitrogen’s Platinum Taq along and dNTPs.

The barcoded libraries were then treated with New England BioProducts Exonuclease I. The 25 µL libraries were incubated with 1.5µl of Exonuclease I for 15 min at 37 °C in a Veriti thermal cycler. The libraries were then manually purified with 1.8× Agencourt Ampure XP (Beckman Coulter, Brea, CA, USA) according to Agencourt/Beckman Coulter’s protocol. The DynaMag-96 Side Magnet was used (Thermo Fisher Scientific). Final libraries were eluted with Qiagen’s Elution Buffer (Buffer EB).

The libraries were then analyzed for quality and quantity using Agilent’s 2100 Bioanalyzer (Agilent, Santa Clara, CA, USA) and Agilent’s DNA 1000 kit. Using Bioanalyzer software version 2100 Expert B.02.08 SI648, region tables were assigned to the libraries, giving approximate sizes and concentrations. Any libraries that failed to amplify well were repeated from the initial PCR step and not included in the final pool until optimal. Each library sample was also quality checked using Thermo Fisher Scientific’s nanodrop ND-8000 spectrophotometer. If required, library samples were normalized on the basis of the lowest concentration from the Bioanalyzer data, and then all libraries were pooled together to that equimolar concentration. The final library pool was then checked one last time for concentration with the ND-8000. 

Using the approximate base pair size and the final nanodrop concentration, the correct load concentration was determined for the library. The library was then processed according to Illumina’s MiSeq System Denature and Dilute Libraries Guide. The control used was PhiX Control kit v.3, (Illumina, San Diego, CA, USA). 

The library was sequenced using the Illumina MiSeq next generation sequencer (Illumina), MiSeq Controller software version 2.6.2.1., using an Illumina MiSeq Reagent Kit v.2 300 Cycle, (Illumina). This was in accordance with the Illumina MiSeq System Guide.

Sample barcode demultiplexed FASTQ files from the MiSeq sequencing were evaluated for the presence of variants of the reference sequence TTTTTGCCATATATATATATATAGTAGGAGAGGGCGAACC. Variants were enumerated by collecting, for each sample, all sequence reads that were bounded by TTTTTGCCA and AGTAGGAGAGGGCGAACC. All variants for each sample were tabulated and sorted according to length and nucleotide sequence. Variant frequency was calculated as the ratio of the number of identical reads for each sample divided by the total number of qualifying reads in the sample.

### 4.3. Fragment Analysis 

Primers were designed for the region of interest within the promoter of UGT1A1, specifically dbSNP ID: rs3064744. This design was a nested PCR, and the outer PCR primers were as follows: FW primer 5′-TTCTTCCTCTCTGGTAACACTT-3′, RV primer 5′-ACTCTTTCACATCCTCCCTT-3′. For the PCR assay, Invitrogen’s High Fidelity Taq System (Thermo Fisher Scientific) and Invitrogen 10 mM dNTPs (Thermo Fisher Scientific) with 5% molecular grade dimethyl sulfoxide (DMSO) (Sigma-Aldrich) were utilized. Samples were amplified with Applied Biosystems Veriti 96-well thermal cycler (Thermo Fisher Scientific) using the following conditions: 95 °C for 5 min; followed by 20 cycles of 94 °C for 1 min, 58 °C for 1 min, 72 °C for 1 min. This was followed by 20 more cycles of 94 °C for 1 min, 65 °C for 1 min, 72 °C for 1 min, with a final extension of 72 °C for 10 min, then holding at 4 °C.

The generated PCR amplicons were then purified using exonuclease I (GE Healthcare, Pittsburgh, PA, USA) and shrimp alkaline phosphatase (Affymetrix), in accordance with the Exo-Sap protocol. The Exo-Sap-sample mixture was then incubated in the Veriti thermal cycler: 37 °C for 15 min, then 80 °C for 15 min, followed by a 4 °C hold. This purified amplicon was then used in the next, inner PCR as described below.

For the inner PCR, the FW PCR primers were 5′-GCTCCACCTTCTTTATCTCTG-3′, 5′-FAM- GCTCCACCTTCTTTATCTCTG-3′, and 5′-GTTTCTGCTCCACCTTCTTTATCTCTG-3′ (pigtailed FW). The RV PCR primers were 5′-ATCAACAGTATCTTCCCAGC-3′, 5′-FAM- ATCAACAGTATCTTCCCAGC-3′, and 5′-GTTTCTATCAACAGTATCTTCCCAGC-3′ (pigtailed RV). Before amplification, primer mixes were created. For the FW FAM-labeled product, a 20 µM FAM-FW primer mix was created by adding 480 µL of molecular grade water, 18 µL of 500 µM RV, and 2 µL of 500 µM FAM-labeled FW primer. The amplification was then carried out with this FAM-FW/RV primer master mix and the pigtailed RV, each at 0.8 µM final concentrations. For the RV FAM-labeled product, a 20 µM FAM-RV primer mix was created by adding 480 µl of molecular grade water, 18 µL of 500 µM FW, and 2 µL of 500 µM FAM-labeled RV primer. The amplification was then carried out with this FAM-RV/FW primer master mix and the pigtailed FW, each at 0.8 µM final concentrations. All samples were tested with both the FAM FW primer master mix and the FAM RV primer master mix for comparison and confirmation. Samples from DNA were amplified for the inner PCR using Platinum Taq and dNTPs, along with the Veriti thermal cycler using the following conditions: 94 °C for 5 min, then 20 cycles of 94 °C for 30 s, 58 °C for 30 s, 72 °C for 30 s; followed by a final extension of 72 °C for 7 min, then holding at 4 °C. 

The resulting products were then checked for quality and concentration with Agilent’s 2100 Bioanalyzer (Agilent) and Agilent’s DNA 1000 kit, using Bioanalyzer software version 2100 Expert B.02.08 SI648. These samples were then diluted with molecular grade water at a 1:10 ratio to prepare them for running on fragment analysis. A master mix was created using Applied Biosystems Hi Di Formamide (Thermo Fisher Scientific) and Applied Biosystems Gene Scan LIZ 500 (Thermo Fisher Scientific). All incubations were carried out with the Veriti thermal cycler. Samples were then processed on Applied Biosystems 3730xl DNA Analyzer (Thermo Fisher Scientific), 96 capillary 50 cm array, using data generated with DS-33 Matrix Standard Kit (Dye Set 5) (Thermo Fisher Scientific). Fragment analysis data was reviewed with Thermo Fisher Scientific Peak Scanner 1.0 software.

### 4.4. Direct Sequencing (in-House)

Primer pairs were designed on the basis of the gene sequence available at Genbank, and the sequences of each primer pair is listed as follows: 5′-AAGCGGGGGTACAGTTGTGTTC-3′, 5′-AAGAATACAGTGGGCAGAGACAG-3′. PCR reactions were carried out in a 50 μL reaction mixture containing 200 ng of genomic DNA, 1X PCR buffer (Thermo Fisher Scientific, Waltham, MA, USA), 1.5 mmol/L MgCl_2_, 0.2 mM deoxynucleotide triphosphates, 800nM of each primer (i.e., F1 and R1), and 1.25 U Platinum Taq DNA polymerase (Invitrogen) using a GeneAmp PCR system 9700 (Thermo Fisher Scientific, Waltham, MA, USA) as a thermocycler with the following thermal profile (primary PCR): 40 cycles of denaturation at 94 °C for 30 s, annealing at 66 °C for 30 s, and extension at 72 °C for 30 s. After amplification, the quality of the amplified PCR products was verified by agarose gel electrophoresis. The PCR products were then sequenced on an ABI Prism 3130xl Genetic Analyzer (Applied Biosystems) per the manufacturer’s instructions using the following sequencing primers: 5′-TCCTTCTTCCTCTCTGGTAAC-3′, 5′-ACATTATGCCCGAGACTAAC-3′.

### 4.5. Pyrosequencing 

Primers were designed for the region of interest within the promoter of UGT1A1, specifically dbSNP ID: rs3064744, and ordered from Invitrogen. The FW Pyromark PCR primer was 5′Bio-CACGTGACACAGTCAAACATTAAC-3′, and the RV PCR primer was 5′- AGGTTCGCCCTCTCCTACTTATA-3′. The Pyromark sequence primer was 5′- CGCCCTCTCCTACTTATATAT-3′ [28]. Assay design scores and primers were checked with Qiagen’s Pyromark Design Software version 2.0. Samples from DNA were amplified using Qiagen’s Pyromark PCR kit (Qiagen) and the listed FW and RV PCR primers, along with the Veriti 96-well thermal cycler (Thermo Fisher Scientific). The following cycling conditions were used: 95 °C for 15 min, then 42 cycles of 95 °C for 20 s, 53 °C for 30 s, 72 °C for 20 s; followed by a final extension of 72 °C for 5 min, then holding at 4 °C.

All amplicons were checked for concentration and quality using Agilent’s 2100 Bioanalyzer (Agilent, Santa Clara, CA, USA) and Agilent’s DNA 1000 kit. Data analysis was performed using Bioanalyzer software version 2100 Expert B.02.08 SI648.

Samples were then processed with Biotage’s MD Pyrosequencing System: Biotage Q96MD, Biotage RIA Vacuum Work Station, and Biotage HS Sample Prep Thermoplate D1200. Qiagen reagents, kits, and consumables were used as listed: Pyromark Q96 HS Plates, Pyromark Gold Q96 Reagents, Pyromark Control Oligo, Pyromark Annealing Buffer, Pyromark Denaturation Buffer, and Pyromark Binding Buffer. Additionally, GE Healthcare’s Streptavidin Sepharose High Performance was used for binding the DNA to the plate. 

Samples were analyzed using Pyromark MD Software version 1.0, a custom dispensation of the sequence listed of was used—GATATATATCATATGCT, with the sequence to analyze being ATATATATGC/TGCAAAAACC/GAATCGATACACCAAGT. However, because this assay design was custom, the calls and analysis for the (TA)_N_ were determined manually and not called by the software.

### 4.6. DMET Plus

Samples were then processed using Applied Biosystems’ DMET Plus Starter kit in accordance with the DMET Plus assay protocol with supplementation of Qiagen’s Multiplex PCR kit for use in the mPCR step of the assay, Titanium Taq DNA Polymerase (Takara Bio, Mountain View, CA, USA) for the amplification stage of the assay step, and Streptavidin R-Phycoerythrin Conjugate (SAPE, Invitrogen) for the preparation of the stain buffer solution used in the wash/stain protocol. All temperature holds and thermal cycling were performed on Applied Biosystems’ GeneAmp PCR System 9700 (Thermo Fisher Scientific). Following the cleanup and fragmentation steps of the assay protocol, sample amplification and fragmentation were assessed using Agilent’s 2100 Bioanalyzer (Agilent) and Agilent’s DNA 1000 kit, software version 2100 Expert B.02.08 SI648. Hybridization occurred in an Affymetrix GeneChip Hybridization Oven 645 (Thermo Fisher Scientific), arrays were registered using Affymetrix GeneChip Command Console (AGCC) Portal version 4.0.0.1567G, wash/stain protocol was performed on an Affymetrix GeneChip Fluidics Station 450 (Thermo Fisher Scientific) using AGCC Fluidics Control software version 4.0.0.1567, and arrays were scanned an Affymetrix GeneChip Scanner 3000 7G Plus (Thermo Fisher Scientific) with an autoloader (Thermo Fisher Scientific) using AGCC Scan Control software version 4.0.0.1567. Array controls were assessed using Affymetrix’s AGCC Viewer software version 4.0.0.1567 and assay results were analyzed on Affymetrix’s DMET Console software version 1.3.0.20. Pharmacoscan was conducted by RUDCR at Rutgers University on a fee-for-service basis.

### 4.7. Statistical Considerations

Hardy–Weinberg equilibrium and comparisons between genotypes and previously published studies were conducted with the chi-square test (or Fisher’s exact test where appropriate). Principal component analyses were conducted to confirm the heuristic groupings of genotypes between the different genotyping methods. Comparisons between previously published studies were conducted using the Fisher–Freeman–Halton test. All comparisons were conducted with Statistical Analysis Software (SAS). 

## Figures and Tables

**Table 1:** Nomenclature and expression level.

Star Nomenclature	(TA)*_n_*	Expression/Function ^*^
UGT1A1*36	5	Highest
UGT1A1*1	6	High
UGT1A1*28	7	Low
UGT1A1*37	8	Lowest

* Expression and function based on [13,14].

**Table 2:** Population by genotype frequency using different genotyping platforms.

	*36/*36	*36/*1	*1/*1	*36/*28	*1/*28	*1/*37	*28/*28	*28/*37	Not Called
Platform	*n* = (%)	*n* = (%)	*n* = (%)	*n* = (%)	*n* = (%)	*n* = (%)	*n* = (%)	*n* = (%)	*n* = (%)
*(TA)n/(TA)n*	5/5	5/6	6/6	5/6	6/7	6/8	7/7	7/8	
*Metabolism Status*	*Normal*			*Intermediate*			*Poor*		
*Illumina (n = 163)*									
Female (*n* = 63)		2 (3.2)	24 (38.1)		29 (46)	1 (1.6)	7 (11.1)		
Male (*n* = 100)			46 (46)	6 (6)	34 (34)		13 (13)	1 (1)	
Caucasian (non-Hispanic) (*n* = 105)			56 (49.1)		48 (42.1)		10 (8.8)		
Black or African American (*n* = 32)		1 (3.1)	7 (21.9)	6 (18.8)	8 (25)	1 (3.1)	8 (25)	1 (3.1)	
Hispanic or Latino (*n* = 5)		1 (20)	2 (40)		2 (40)				
Asian or Pacific Islander (*n* = 9)			5 (55.6)		4 (44.4)				
Other or Unknown (*n* = 3)					1 (33.3)		2 (66.7)		
*Fragment Analysis (n = 163)*									
Female (*n* = 63)		2 (3.2)	24 (38.1)		29 (46)	1 (1.6)	7 (11.1)		
Male (*n* = 100)			46 (46)	6 (6)	34 (34)		13 (13)	1 (1)	
Caucasian (non-Hispanic) (*n* = 105)			56 (49.1)		48 (42.1)		10 (8.8)		
Black or African American (*n* = 32)		1 (3.1)	7 (21.9)	6 (18.8)	8 (25)	1 (3.1)	8 (25)	1 (3.1)	
Hispanic or Latino (*n* = 5)		1 (20)	2 (40)		2 (40)				
Asian or Pacific Islander (*n* = 9)			5 (55.6)		4 (44.4)				
Other or Unknown (*n* = 3)					1 (33.3)		2 (66.7)		
*fPCR (n =* 9) ^1^									
Female (*n* = 6)		2 (33.3)	1 (16.7)		1 (16.7)	1 (16.7)	1 (16.7)		
Male (*n* = 7)			1 (14.3)	2 (28.6)	2 (28.6)		1 (14.3)	1 (14.3)	
Caucasian (non-Hispanic) (*n* = 6)			2 (33.3)		2 (33.3)		2 (33.3)		
Black or African American (*n* = 6)		1 (16.7)		2 (33.3)	1 (16.7)	1 (16.7)		1 (16.7)	
Hispanic or Latino (*n* = 1)		1 (100)							
*Pyrosequencing (n = 162)*									
Female (*n* = 63)		2 (3.2)	23 (36.5)		29 (46)		6 (9.5)		3 (4.8)
Male (*n* = 99)			45 (45.5)	5 (5.1)	32 (32.3)		13 (13.1)		4 (4)
Caucasian (non-Hispanic) (*n* = 113)			54 (47.8)		47 (41.6)		9 (8)		3 (2.7)
Black or African American (*n* = 32)		1 (3.1)	7 (21.9)	5 (15.6)	7 (21.9)		8 (25)		4 (12.5)
Hispanic or Latino (*n* = 5)		1 (20)	2 (40)		2 (40)				
Asian or Pacific Islander (*n* = 9)			5 (55.6)		4 (44.4)				
Other or Unknown (*n* = 3)					1 (33.3)		2 (66.7)		
*Pyromark Gel (n = 162)*									
Female (*n* = 63)		2 (3.2)	24 (38.1)		29 (46)		7 (11.1)		1 (1.6)
Male (*n* = 99)			46 (46.5)	5 (5.1)	33 (33.3)		13 (13.1)		2 (2)
Caucasian (non-Hispanic) (*n* = 113)			56 (49.6)		47 (41.6)		10 (8.8)		
Black or African American (*n* = 32)		1 (3.1)	7 (21.9)	5 (15.6)	8 (25)		8 (25)		3 (9.4)
Hispanic or Latino (*n* = 5)		1 (20)	2 (40)		2 (40)				
Asian or Pacific Islander (*n* = 9)			5 (55.6)		4 (44.4)				
Other or Unknown (*n* = 3)					1 (33.3)		2 (66.7)		
*DMET* (*n =* 168) ^2^									
Female (*n* = 65)	28 (43.1)			30 (46.2)			7 (10.8)		
Male (*n* = 103)	55 (53.4)			34 (33)			14 (13.6)		
Caucasian (non-Hispanic) (*n* = 117)	60 (51.3)			47 (40.2)			10 (8.5)		
Black or African American (*n* = 32)	13 (40.6)			10 (31.3)			9 (28.1)		
Hispanic or Latino (*n* = 5)	3 (60)			2 (40)					
Asian or Pacific Islander (*n* = 11)	7 (63.6)			4 (36.4)					
Other or Unknown (*n* = 3)				1 (33.3)			2 (66.7)		
*Pharmacoscan (n =* 21) ^1,3^									
Female (*n* = 5)		1 (20)	2 (40)		1 (20)		1 (20)		
Male (*n* = 16)			2 (12.5)	5 (31.3)	6 (37.5)		3 (18.8)		
Caucasian (non-Hispanic) (*n* = 11)			3 (27.3)		5 (45.5)		3 (27.3)		
Black or African American (*n* = 9)			1 (11.1)	5 (55.6)	2 (22.2)		1 (11.1)		
Hispanic or Latino (*n* = 1)		1 (100)							

^1^ Selected samples were used to confirm representative genotype results from other assays. Hence, genotype frequency does not reflect the actual population. ^2^ The assay only indicates whether a patient carries (TA)_5_ or (TA)_6_ or the patient carries (TA)_7_ or (TA)_8_. Thus, metabolism status is known, but specific genotypes are not provided. ^3^ The assay only indicates whether a patient carries (TA)_7_ or (TA)_8_. fPCR: fluorescent PCR.

**Table 3:** Concordance rate among different genotyping platforms.

	Illumina Fraction (%)	Fragment Analysis Fraction (%)	DMET Plus Fraction (%)	Pyrosequencing Fraction (%)
*Miscalls only*				
Illumina (*n =* 163)	x	163/163 (100)	154/163 (94.5)	155/155 (100)
Fragment Analysis (*n =* 163)		x	154/163 (94.5)	155/155 (100)
DMET Plus (*n =* 168)			x	146/155 (94.2)
Pyrosequencing (n = 155) *				x
*Miscalls and ambiguous calls*				
Illumina (*n =* 163)	x	163/163 (100)	159/163 (97.5)	155/162 (95.7)
Fragment Analysis (*n =* 163)		x	159/163 (97.5)	155/162 (95.7)
DMET Plus (*n =* 168)			x	146/162 (90.1)
Pyrosequencing (*n* = 162) *				x

* Several ambiguous calls were noted for pyrosequencing, resulting in a change from n = 155 to n = 162.

## Supplementary Materials

Supplementary materials can be found at https://www.mdpi.com/1422-0067/21/3/896/s1.
