# Supplementary material for: Comparison of Eight Technologies to Determine Genotype at the UGT1A1 (TA)n Repeat Polymorphism: Potential Clinical Consequences of Genotyping Errors?
Source: Int J Mol Sci. 2020 Jan 30;21(3):896. doi: 10.3390/ijms21030896 (PMC7037496; doi:10.3390/ijms21030896)
Supplement: Supplementary file 1 [file ijms-21-00896-s001.zip › TABLE S3.docx]

**TABLE S2.** Fragment analysis genotypes vs vendor-reported genotype

| Controls | Genotype per the vendor | (TA)_n_/(TA)_n_ per the vendor | Fragment Analysis Genotype | (TA)_n_/(TA)_n_ by Fragment Analysis |
| --- | --- | --- | --- | --- |
| NA10854 | **1/*1* | 6/6 | **1/*1* | 6/6 |
| NA11993 | **1/*28* | 6/7 | **1/*28* | 6/7 |
| NA10831 | **28/*28* | 7/7 | **28/*28* | 7/7 |
| NA19213 | **36/*1* | 5/6 | **36/*1* | 5/6 |
