# Supplementary material for: Comparison of Eight Technologies to Determine Genotype at the UGT1A1 (TA)n Repeat Polymorphism: Potential Clinical Consequences of Genotyping Errors?
Source: Int J Mol Sci. 2020 Jan 30;21(3):896. doi: 10.3390/ijms21030896 (PMC7037496; doi:10.3390/ijms21030896)
Supplement: Supplementary file 1 [file ijms-21-00896-s001.zip › Figure S2.pdf]

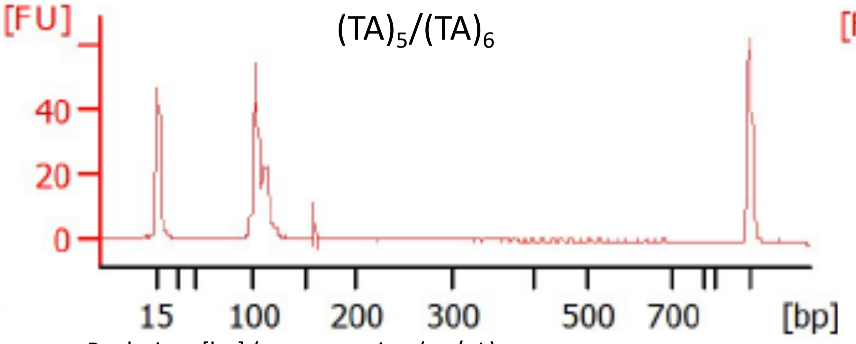

Peak sizes [bp] (concentration (ng/μL):
15 (4.20), 104 (3.70), 113 (2.22), 1500 (2.10)

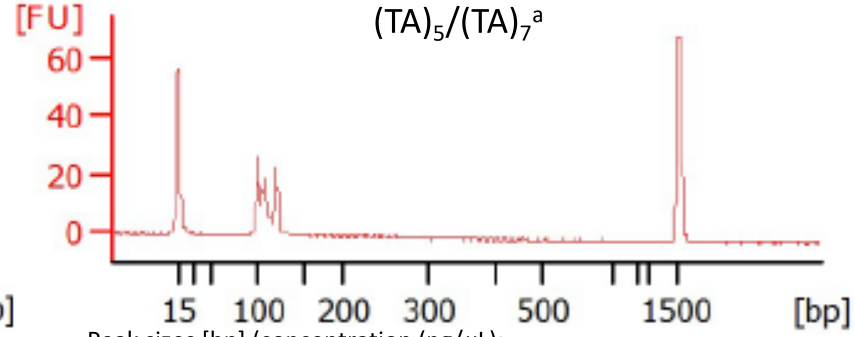

Peak sizes [bp] (concentration (ng/μL):
15 (4.20), 102 (1.50), 121 (1.22), 1500 (2.10)

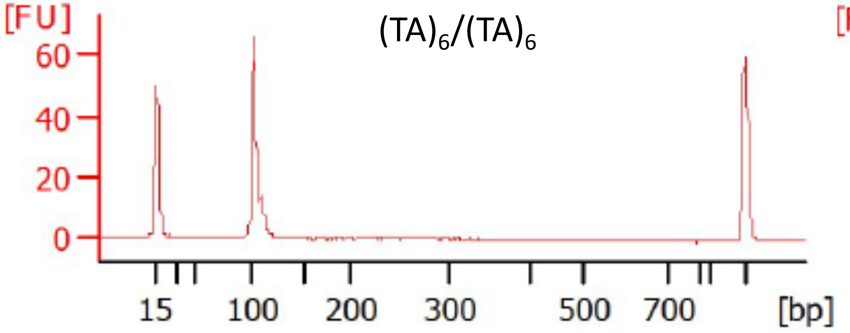

Peak sizes [bp] (concentration (ng/μL):
15 (4.20), 104 (3.99), 1500 (2.10)

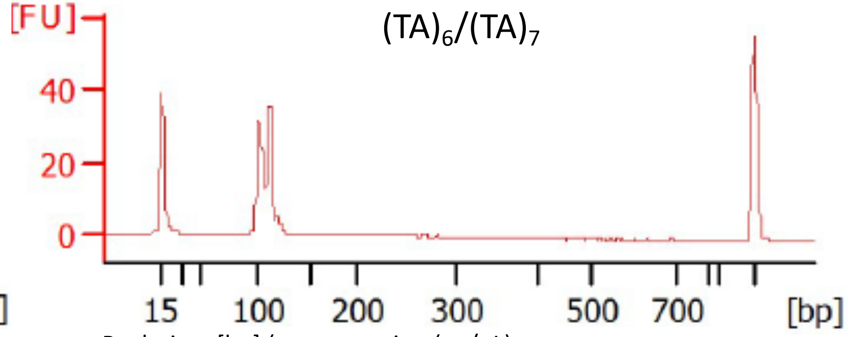

Peak sizes [bp] (concentration (ng/μL):
15 (4.20), 103 (3.29), 113 (2.41), 1500 (2.10)

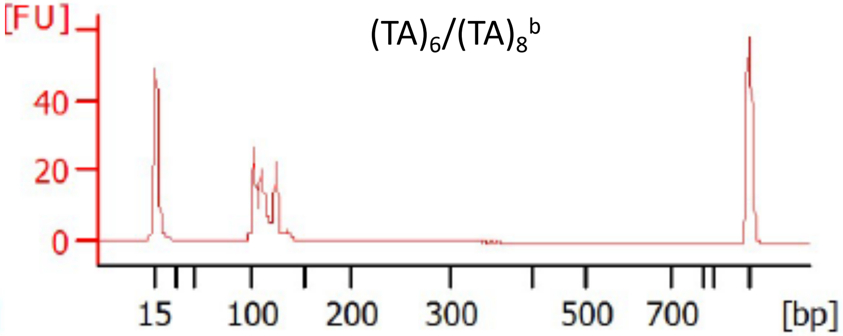

Peak sizes [bp] (concentration (ng/μL):
15 (4.20), 104 (1.75), 111 (1.88), 124 (1.41), 1500 (2.10)

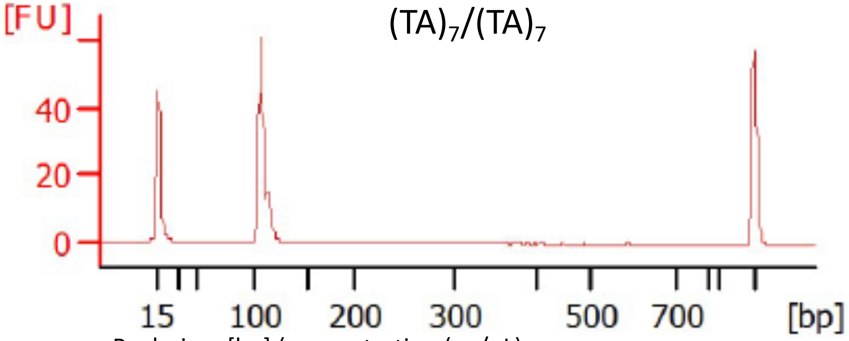

Peak sizes [bp] (concentration (ng/μL):
15 (4.20), 108 (3.99), 1500 (2.10)

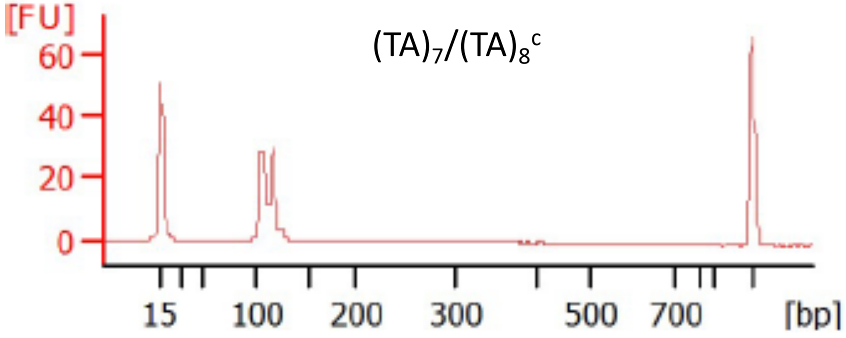

Peak sizes [bp] (concentration (ng/μL):
15 (4.20), 105 (2.49), 115 (1.84), 1500 (2.10)
