# Supplementary material for: Comparison of Eight Technologies to Determine Genotype at the UGT1A1 (TA)n Repeat Polymorphism: Potential Clinical Consequences of Genotyping Errors?
Source: Int J Mol Sci. 2020 Jan 30;21(3):896. doi: 10.3390/ijms21030896 (PMC7037496; doi:10.3390/ijms21030896)
Supplement: Supplementary file 1 [file ijms-21-00896-s001.zip › TABLE S4.docx]

**TABLE S3.** Comparison of previously published studies utilizing direct sequencing, fragment analysis, gel sizing, or pyrosequencing.

| Race | Disease or Control? | Method | n total= | (TA)5/(TA)5 | (TA)5/(TA)6 | (TA)5/(TA)7 | (TA)5/(TA)8 | (TA)6/(TA)6 | (TA)6/(TA)7 | (TA)6/(TA)8 | (TA)7/(TA)7 | (TA)7/(TA)8 | (TA)8/(TA)8 | P alleles (TA)6 | Q alleles (TA)5 | Q alleles (TA)7 | Q alleles (TA)8 | Total alleles | PubMed ID# |
| --- | --- | --- | --- | --- | --- | --- | --- | --- | --- | --- | --- | --- | --- | --- | --- | --- | --- | --- | --- |
| African American | Repository Samples | Direct Sequencing | 119 | 0 | 5 | 8 | 2 | 32 | 48 | 6 | 14 | 4 | 0 | 123 | 15 | 88 | 12 | 238 | 17459361 |
| African American | Sickle Cell | Direct Sequencing | 201 | 1 | 22 | 10 | 0 | 56 | 66 | 1 | 39 | 6 | 0 | 201 | 34 | 160 | 7 | 402 | 15388579 |
| African American | Control | Direct Sequencing | 111 | 0 | 6 | 6 | 0 | 24 | 50 | 3 | 17 | 4 | 1 | 107 | 12 | 94 | 9 | 222 | 15388579 |
| Black (Carribean) | Healthy | Direct Sequencing | 570 | 6 | 33 | 33 | 2 | 131 | 236 | 21 | 91 | 17 | 0 | 552 | 80 | 468 | 40 | 1140 | 27074016 |
| Black (Carribean) | Prostate Cancer | Direct Sequencing | 549 | 6 | 27 | 26 | 5 | 146 | 185 | 26 | 108 | 19 | 1 | 530 | 70 | 446 | 52 | 1098 | 27074016 |
| Black (Central Africa) | Healthy | Direct Sequencing | 138 | 0 | 11 | 15 | 3 | 27 | 42 | 3 | 29 | 8 | 0 | 110 | 29 | 123 | 14 | 276 | 27074016 |
| Black (Central Africa) | Prostate Cancer | Direct Sequencing | 147 | 1 | 10 | 7 | 1 | 34 | 53 | 4 | 29 | 7 | 1 | 135 | 20 | 125 | 14 | 294 | 27074016 |
| Caucasian | Cancer | Direct Sequencing | 93 | 0 | 0 | 0 | 0 | 37 | 45 | 0 | 11 | 0 | 0 | 119 | 0 | 67 | 0 | 186 | 17611564 |
| Caucasian | Control | Direct Sequencing | 202 | 0 | 2 | 0 | 0 | 102 | 75 | 1 | 22 | 0 | 0 | 282 | 2 | 119 | 1 | 404 | 10471066 |
| Caucasian | Volunteer | Direct Sequencing | 1091 | 0 | 0 | 0 | 0 | 509 | 485 | 0 | 97 | 0 | 0 | 1503 | 0 | 679 | 0 | 2182 | 19389676 |
| Caucasian | Volunteer | Direct Sequencing | 11 | 0 | 0 | 0 | 0 | 6 | 4 | 0 | 1 | 0 | 0 | 16 | 0 | 6 | 0 | 22 | 20823282 |
| Caucasian |  | Direct Sequencing | 12 | 0 | 0 | 0 | 0 | 6 | 5 | 0 | 1 | 0 | 0 | 17 | 0 | 7 | 0 | 24 | 29389076 |
| Caucasian (150/163) | Volunteer | Direct Sequencing | 163 | 0 | 0 | 0 | 0 | 80 | 72 | 0 | 11 | 0 | 0 | 232 | 0 | 94 | 0 | 326 | 15318931 |
| Caucasian (93%) | Volunteer | Direct Sequencing | 100 | 0 | 0 | 0 | 0 | 37 | 54 | 0 | 9 | 0 | 0 | 128 | 0 | 72 | 0 | 200 | 16771603 |
| Caucasian (Italian) | Healthy | Direct Sequencing | 70 | 0 | 0 | 0 | 0 | 26 | 30 | 0 | 14 | 0 | 0 | 82 | 0 | 58 | 0 | 140 | 24726540 |
| Caucasian (Norway) | Cancer | Direct sequencing | 70 | 0 | 0 | 0 | 0 | 32 | 33 | 0 | 5 | 0 | 0 | 97 | 0 | 43 | 0 | 140 | 12185559 |
| Caucasian (Polish) | Beta-Thalassaemia | Direct Sequencing | 137 | 0 | 0 | 0 | 0 | 57 | 55 | 0 | 25 | 0 | 0 | 169 | 0 | 105 | 0 | 274 | 22554963 |
| Caucasian (Polish) | Glucose-6-Phosphate Dehydrogease Deficiency | Direct Sequencing | 54 | 0 | 1 | 0 | 0 | 11 | 29 | 0 | 12 | 1 | 0 | 52 | 1 | 54 | 1 | 108 | 22554963 |
| Caucasian (Polish) | Healthy | Direct Sequencing | 182 | 0 | 0 | 1 | 0 | 66 | 82 | 0 | 33 | 0 | 0 | 214 | 1 | 149 | 0 | 364 | 22554963 |
| Caucasian (Polish) | Hereditary Spherocytosis | Direct Sequencing | 65 | 0 | 0 | 2 | 0 | 24 | 23 | 0 | 16 | 0 | 0 | 71 | 2 | 57 | 0 | 130 | 22554963 |
| Caucasian (Slovenian) | Volunteer | Direct Sequencing | 236 | 0 | 0 | 0 | 0 | 90 | 113 | 0 | 32 | 1 | 0 | 293 | 0 | 178 | 1 | 472 | 17196409 |
| Caucasian (Spanish) | Acromegaly | Direct Sequencing | 36 | 0 | 0 | 0 | 0 | 15 | 16 | 0 | 5 | 0 | 0 | 46 | 0 | 26 | 0 | 72 | 20207827 |
| Caucasian (Spanish) | Cancer | Direct Sequencing | 149 | 0 | 0 | 0 | 0 | 56 | 78 | 0 | 15 | 0 | 0 | 190 | 0 | 108 | 0 | 298 | 20628391 |
| Caucasian (Spanish) | Patients Receiving SN-38 | Direct Sequencing | 59 | 0 | 0 | 0 | 0 | 32 | 23 | 0 | 4 | 0 | 0 | 87 | 0 | 31 | 0 | 118 | 23789755 |
| Caucasian (USA) | mCRPC | Direct Sequencing | 100 | 0 | 0 | 0 | 0 | 64 | 27 | 0 | 9 | 0 | 0 | 155 | 0 | 45 | 0 | 200 | 26108357 |
| African | Repository Samples | Fragment Analysis | 101 | 0 | 2 | 5 | 0 | 26 | 37 | 4 | 19 | 6 | 2 | 95 | 7 | 86 | 14 | 202 | 18043502 |
| African (Nigeria) | Cancer | Fragment Analysis | 502 | 10 | 38 | 36 | 13 | 100 | 177 | 20 | 90 | 15 | 3 | 435 | 107 | 408 | 54 | 1004 | 17909964 |
| African (Nigeria) | Control | Fragment Analysis | 222 | 2 | 26 | 15 | 1 | 28 | 88 | 5 | 45 | 11 | 1 | 175 | 46 | 204 | 19 | 444 | 17909964 |
| African (Nigerian) | Repository Samples | Fragment Analysis | 88 | 1 | 5 | 5 | 0 | 19 | 38 | 1 | 16 | 3 | 0 | 82 | 12 | 78 | 4 | 176 | 18043502 |
| African American | Breast Cancer | Fragment Analysis | 200 | 2 | 17 | 14 | 2 | 46 | 73 | 3 | 38 | 5 | 0 | 185 | 37 | 168 | 10 | 400 | 10706110 |
| African American | Cancer | Fragment Analysis | 25 | 0 | 4 | 2 | 0 | 5 | 10 | 1 | 2 | 1 | 0 | 25 | 6 | 17 | 2 | 50 | 12969965 |
| African American | Cancer | Fragment Analysis | 25 | 0 | 2 | 4 | 1 | 8 | 5 | 2 | 1 | 2 | 0 | 25 | 7 | 13 | 5 | 50 | 16280036 |
| African American | Cancer | Fragment Analysis | 11 | 0 | 0 | 0 | 0 | 5 | 5 | 0 | 1 | 0 | 0 | 15 | 0 | 7 | 0 | 22 | 19349540 |
| African American | Cancer | Fragment Analysis | 11 | 0 | 0 | 0 | 0 | 5 | 5 | 0 | 1 | 0 | 0 | 15 | 0 | 7 | 0 | 22 | 19349540 |
| African American | Control | Fragment Analysis | 200 | 1 | 19 | 10 | 0 | 56 | 72 | 4 | 33 | 5 | 0 | 207 | 31 | 153 | 9 | 400 | 10706110 |
| African American | Control | Fragment Analysis | 39 | 0 | 0 | 2 | 1 | 10 | 13 | 6 | 5 | 2 | 0 | 39 | 3 | 27 | 9 | 78 | 12464801 |
| African American | Repository Samples | Fragment Analysis | 119 | 0 | 5 | 8 | 2 | 32 | 48 | 6 | 14 | 4 | 0 | 123 | 15 | 88 | 12 | 238 | 17459361 |
| African American | Repository Samples | Fragment Analysis |  |  |  |  |  |  |  |  |  |  |  | 349 | 42 | 251 | 28 | 670 | 17478602 |
| Caucasian | Cancer | Fragment Analysis | 77 | 0 | 0 | 0 | 0 | 35 | 34 | 0 | 8 | 0 | 0 | 104 | 0 | 50 | 0 | 154 | 12969965 |
| Caucasian | Cancer | Fragment Analysis | 173 | 2 | 1 | 0 | 0 | 67 | 86 | 0 | 17 | 0 | 0 | 221 | 5 | 120 | 0 | 346 | 16280036 |
| Caucasian | Cancer | Fragment Analysis | 67 | 0 | 0 | 0 | 0 | 31 | 26 | 0 | 10 | 0 | 0 | 88 | 0 | 46 | 0 | 134 | 19349540 |
| Caucasian | Cancer | Fragment Analysis | 542 | 0 | 0 | 0 | 0 | 247 | 245 | 0 | 50 | 0 | 0 | 739 | 0 | 345 | 0 | 1084 | 19352303 |
| Caucasian | Cancer | Fragment Analysis | 67 | 0 | 0 | 0 | 0 | 31 | 26 | 0 | 10 | 0 | 0 | 88 | 0 | 46 | 0 | 134 | 19349540 |
| Caucasian | Control | Fragment Analysis | 56 | 0 | 1 | 0 | 0 | 21 | 26 | 0 | 7 | 1 | 0 | 69 | 1 | 41 | 1 | 112 | 12464801 |
| Caucasian | Control | Fragment Analysis | 605 | 0 | 0 | 0 | 0 | 281 | 255 | 0 | 69 | 0 | 0 | 817 | 0 | 393 | 0 | 1210 | 18790042 |
| Caucasian | Control | Fragment Analysis | 198 | 0 | 0 | 0 | 0 | 60 | 92 | 0 | 46 | 0 | 0 | 212 | 0 | 184 | 0 | 396 | 19309288 |
| Caucasian | Control | Fragment Analysis | 1390 | 0 | 0 | 0 | 0 | 628 | 596 | 0 | 166 | 0 | 0 | 1852 | 0 | 928 | 0 | 2780 | 19352303 |
| Caucasian | High cholesterol | Fragment Analysis | 464 | 0 | 0 | 0 | 0 | 218 | 197 | 0 | 49 | 0 | 0 | 633 | 0 | 295 | 0 | 928 | 18790042 |
| Caucasian | Liver Donors | Fragment Analysis | 53 | 0 | 0 | 0 | 0 | 21 | 25 | 0 | 7 | 0 | 0 | 67 | 0 | 39 | 0 | 106 | 17622938 |
| Caucasian | Repository Samples | Fragment Analysis |  |  |  |  |  |  |  |  |  |  |  | 245 | 0 | 117 | 0 | 362 | 17478602 |
| Caucasian | Volunteer | Fragment Analysis | 43 | 0 | 0 | 0 | 0 | 15 | 15 | 0 | 13 | 0 | 0 | 45 | 0 | 41 | 0 | 86 | 17374650 |
| Caucasian |  | Fragment Analysis | 3245 | 0 | 0 | 0 | 0 | 1370 | 1473 | 0 | 402 | 0 | 0 | 4213 | 0 | 2277 | 0 | 6490 | 29220881 |
| Caucasian (American) | Volunteer | Fragment Analysis | 56 | 1 | 0 | 0 | 0 | 26 | 22 | 0 | 7 | 0 | 0 | 74 | 2 | 36 | 0 | 112 | 17762398 |
| Caucasian (Canada) | Bladder Cancer | Fragment analysis | 185 | 0 | 0 | 0 | 0 | 98 | 73 | 0 | 14 | 0 | 0 | 269 | 0 | 101 | 0 | 370 | 26645279 |
| Caucasian (Croatia) | Healthy | Fragment Analysis | 187 | 0 | 0 | 0 | 0 | 83 | 80 | 0 | 24 | 0 | 0 | 246 | 0 | 128 | 0 | 374 | 23981182 |
| Caucasian (Czech) | Chronic Staple Plaque Psoriasis | Fragment Analysis | 46 | 0 | 0 | 0 | 0 | 24 | 19 | 0 | 0 | 0 | 3 | 67 | 0 | 19 | 6 | 92 | 27188524 |
| Caucasian (Czech) | Crohn's Diease | Fragment Analysis | 623 | 0 | 0 | 0 | 0 | 246 | 294 | 0 | 83 | 0 | 0 | 786 | 0 | 460 | 0 | 1246 | 24407487 |
| Caucasian (Czech) | Healthy | Fragment Analysis | 370 | 0 | 0 | 0 | 0 | 141 | 171 | 0 | 58 | 0 | 0 | 453 | 0 | 287 | 0 | 740 | 24407487 |
| Caucasian (Czechia) | Colorectal Cancer | Fragment Analysis | 777 | 0 | 0 | 0 | 0 | 348 | 324 | 0 | 105 | 0 | 0 | 1020 | 0 | 534 | 0 | 1554 | 22212955 |
| Caucasian (Czechia) | Healthy | Fragment Analysis | 986 | 0 | 0 | 0 | 0 | 388 | 453 | 0 | 145 | 0 | 0 | 1229 | 0 | 743 | 0 | 1972 | 22212955 |
| Caucasian (German) | Cancer | Fragment analysis | 103 | 0 | 0 | 0 | 0 | 41 | 52 | 0 | 10 | 0 | 0 | 134 | 0 | 72 | 0 | 206 | 19859999 |
| Caucasian (Greek) | BHP | Fragment Analysis | 138 | 0 | 0 | 0 | 0 | 50 | 58 | 0 | 30 | 0 | 0 | 158 | 0 | 118 | 0 | 276 | 24057187 |
| Caucasian (Greek) | Cancer | Fragment Analysis | 120 | 0 | 0 | 0 | 0 | 45 | 49 | 0 | 26 | 0 | 0 | 139 | 0 | 101 | 0 | 240 | 20308029 |
| Caucasian (Greek) | Control | Fragment Analysis | 256 | 0 | 0 | 0 | 0 | 112 | 106 | 0 | 38 | 0 | 0 | 330 | 0 | 182 | 0 | 512 | 20308029 |
| Caucasian (Greek) | Healthy | Fragment Analysis | 283 | 0 | 0 | 0 | 0 | 110 | 123 | 0 | 50 | 0 | 0 | 343 | 0 | 223 | 0 | 566 | 24057187 |
| Caucasian (Italian) | HBV/HCV-Positive | Fragment Analysis | 167 | 0 | 0 | 0 | 0 | 66 | 80 | 0 | 21 | 0 | 0 | 212 | 0 | 122 | 0 | 334 | 28294511 |
| Caucasian (Italian) | Healthy | Fragment Analysis | 192 | 0 | 0 | 0 | 0 | 69 | 90 | 0 | 33 | 0 | 0 | 228 | 0 | 156 | 0 | 384 | 28294511 |
| Caucasian (Italian) | Hepatocellular Carcinoma | Fragment Analysis | 192 | 0 | 0 | 0 | 0 | 97 | 77 | 0 | 18 | 0 | 0 | 271 | 0 | 113 | 0 | 384 | 28294511 |
| Caucasian (primarily) | Breast Cancer | Fragment Analysis | 455 | 0 | 0 | 0 | 0 | 218 | 196 | 0 | 41 | 0 | 0 | 632 | 0 | 278 | 0 | 910 | 11401924 |
| Caucasian (primarily) | Cancer | Fragment analysis | 92 | 0 | 0 | 0 | 0 | 43 | 41 | 0 | 8 | 0 | 0 | 127 | 0 | 57 | 0 | 184 | 19450125 |
| Caucasian (primarily) | Control | Fragment Analysis | 608 | 0 | 0 | 0 | 0 | 276 | 272 | 0 | 60 | 0 | 0 | 824 | 0 | 392 | 0 | 1216 | 11401924 |
| Caucasian (Sweden) | Control | Fragment Analysis | 1075 | 0 | 1 | 3 | 0 | 508 | 464 | 0 | 98 | 1 | 0 | 1481 | 4 | 664 | 1 | 2150 | 20562445 |
| Caucasian (Sweden) | Control | Fragment Analysis | 423 | 0 | 3 | 0 | 0 | 219 | 159 | 0 | 42 | 0 | 0 | 600 | 3 | 243 | 0 | 846 | 20948202 |
| Caucasian (Sweden) | Myocardial Infarction | Fragment Analysis | 571 | 0 | 0 | 0 | 0 | 270 | 247 | 0 | 54 | 0 | 0 | 787 | 0 | 355 | 0 | 1142 | 20562445 |
| Caucasian (Sweden) | Myocardial Infarction | Fragment Analysis | 215 | 0 | 1 | 0 | 0 | 101 | 90 | 0 | 23 | 0 | 0 | 293 | 1 | 136 | 0 | 430 | 20948202 |
| Caucasians (Italian) | Cancer | Fragment Analysis | 160 | 0 | 0 | 0 | 0 | 69 | 66 | 0 | 25 | 0 | 0 | 204 | 0 | 116 | 0 | 320 | 15254716 |
| Sephardic Jewish | Repository Samples | Fragment Analysis | 262 | 0 | 0 | 0 | 0 | 116 | 110 | 0 | 36 | 0 | 0 | 342 | 0 | 182 | 0 | 524 | 18043502 |
| Caucasian | Cancer | Gel Sizing | 95 | 0 | 0 | 0 | 0 | 40 | 45 | 0 | 10 | 0 | 0 | 125 | 0 | 65 | 0 | 190 | 15280927 |
| Caucasian | Cancer | Gel Sizing | 107 | 0 | 0 | 0 | 0 | 46 | 50 | 0 | 11 | 0 | 0 | 142 | 0 | 72 | 0 | 214 | 18347181 |
| Caucasian | Cancer | Gel Sizing | 18 | 0 | 0 | 0 | 0 | 8 | 6 | 0 | 4 | 0 | 0 | 22 | 0 | 14 | 0 | 36 | 11990381 |
| Caucasian | Cancer | Gel Sizing | 95 | 0 | 0 | 0 | 0 | 36 | 48 | 0 | 11 | 0 | 0 | 120 | 0 | 70 | 0 | 190 | 14744740 |
| Caucasian | Cancer | Gel Sizing | 58 | 0 | 0 | 0 | 0 | 34 | 22 | 0 | 2 | 0 | 0 | 90 | 0 | 26 | 0 | 116 | 12960109 |
| Caucasian (Dutch) | Cancer | Gel Sizing | 371 | 0 | 0 | 0 | 0 | 175 | 159 | 0 | 37 | 0 | 0 | 509 | 0 | 233 | 0 | 742 | 15319294 |
| Caucasian (Dutch) | Cancer | Gel Sizing | 399 | 0 | 0 | 0 | 0 | 183 | 169 | 0 | 47 | 0 | 0 | 535 | 0 | 263 | 0 | 798 | 15319294 |
| Caucasian (Dutch) | Control | Gel Sizing | 253 | 0 | 0 | 0 | 0 | 105 | 116 | 0 | 32 | 0 | 0 | 326 | 0 | 180 | 0 | 506 | 12480553 |
| Caucasian (Greek children) | Control | Gel Sizing | 70 | 0 | 0 | 0 | 0 | 34 | 23 | 0 | 13 | 0 | 0 | 91 | 0 | 49 | 0 | 140 | 11079211 |
| Caucasian (Greek women) | Cancer | Gel Sizing | 136 | 0 | 0 | 0 | 0 | 54 | 56 | 0 | 26 | 0 | 0 | 164 | 0 | 108 | 0 | 272 | 17949292 |
| Caucasian (Greek women) | Control | Gel Sizing | 186 | 0 | 0 | 0 | 0 | 64 | 95 | 0 | 27 | 0 | 0 | 223 | 0 | 149 | 0 | 372 | 17949292 |
| Caucasian (Netherlands) | Healthy | Gel Sizing | 417 | 0 | 0 | 0 | 0 | 172 | 195 | 0 | 50 | 0 | 0 | 539 | 0 | 295 | 0 | 834 | 21351260 |
| Caucasian (Netherlands) | Squamous Cell Carcinoma of the Head and Neck | Gel Sizing | 421 | 0 | 0 | 0 | 0 | 207 | 177 | 0 | 37 | 0 | 0 | 591 | 0 | 251 | 0 | 842 | 21351260 |
| Caucasian (Portuguese) | End-Stage Renal Disease | Gel Sizing | 191 | 0 | 0 | 0 | 0 | 94 | 81 | 16 | 0 | 0 | 0 | 285 | 0 | 81 | 16 | 382 | 25276769 |
| Caucasian (Romaian) | Healthy | Gel Sizing | 605 | 0 | 0 | 11 | 0 | 66 | 517 | 11 | 0 | 0 | 0 | 660 | 11 | 528 | 11 | 1210 | 28338110 |
| Caucasian (Scottish) | Control | Gel Sizing | 77 | 0 | 0 | 0 | 0 | 31 | 37 | 0 | 9 | 0 | 0 | 99 | 0 | 55 | 0 | 154 | 8596320 |
| Caucasians (German) | Control | Gel Sizing | 303 | 0 | 1 | 0 | 0 | 127 | 139 | 0 | 36 | 0 | 0 | 394 | 1 | 211 | 0 | 606 | 12078936 |
| Predominantly Caucasian (British) |  | Gel Sizing | 2190 | 0 | 7 | 2 | 0 | 1079 | 899 | 1 | 202 | 0 | 0 | 3065 | 9 | 1305 | 1 | 4380 | 25086287 |
| African American | Cancer | Pyrosequencing |  |  |  |  |  |  |  |  |  |  |  | 46 | 12 | 47 | 5 | 110 | 16985250 |
| African American | Control | Pyrosequencing |  |  |  |  |  |  |  |  |  |  |  | 318 | 41 | 257 | 32 | 648 | 16985250 |
| Caucasian | Cancer | Pyrosequencing |  |  |  |  |  |  |  |  |  |  |  | 598 | 5 | 291 | 0 | 894 | 16985250 |
| Caucasian | Cancer | Pyrosequencing | 250 | 0 | 0 | 0 | 0 | 114 | 114 | 0 | 22 | 0 | 0 | 342 | 0 | 158 | 0 | 500 | 19364970 |
| Caucasian | Control | Pyrosequencing |  |  |  |  |  |  |  |  |  |  |  | 1321 | 10 | 669 | 4 | 2004 | 16985250 |
| Caucasian | High cholesterol | Pyrosequencing | 35 | 0 | 0 | 0 | 0 | 14 | 18 | 0 | 3 | 0 | 0 | 46 | 0 | 24 | 0 | 70 | 18551036 |
| Caucasian (Dutch) | Cancer | Pyrosequencing |  |  |  |  |  |  |  |  |  |  |  | 43 | 0 | 13 | 0 | 56 | 15122075 |
| Caucasian (Dutch) | Cancer | Pyrosequencing | 147 | 0 | 0 | 0 | 0 | 74 | 63 | 0 | 10 | 0 | 0 | 211 | 0 | 83 | 0 | 294 | 17185998 |
| Caucasian (European, primarily CAUC) | Cancer | Pyrosequencing | 58 | 0 | 0 | 0 | 0 | 34 | 22 | 0 | 2 | 0 | 0 | 44 | 0 | 14 | 0 | 58 | 12960109 |
| Caucasian (French) | Cancer | Pyrosequencing | 75 | 0 | 1 | 1 | 0 | 31 | 35 | 0 | 7 | 0 | 0 | 98 | 2 | 50 | 0 | 150 | 15297419 |
| Caucasian (German, not completely Cauc) | Unclear | Pyrosequencing | 115 | 0 | 1 | 1 | 0 | 49 | 49 | 0 | 15 | 0 | 0 | 148 | 2 | 80 | 0 | 230 | 15049432 |
| Caucasian (Italian) | HIV | Pyrosequencing | 46 | 0 | 0 | 0 | 0 | 21 | 19 | 0 | 6 | 0 | 0 | 61 | 0 | 31 | 0 | 92 | 22661571 |
| Caucasian (Nordic) | Healthy | Pyrosequencing | 148 | 0 | 0 | 0 | 0 | 64 | 79 | 0 | 5 | 0 | 0 | 207 | 0 | 89 | 0 | 296 | 24587300 |
