# Supplementary material for: Comparison of Eight Technologies to Determine Genotype at the UGT1A1 (TA)n Repeat Polymorphism: Potential Clinical Consequences of Genotyping Errors?
Source: Int J Mol Sci. 2020 Jan 30;21(3):896. doi: 10.3390/ijms21030896 (PMC7037496; doi:10.3390/ijms21030896)
Supplement: Supplementary file 1 [file ijms-21-00896-s001.zip › TABLE S1.docx]

| Company/Organization | Test Code | Target | Method | URL |
| --- | --- | --- | --- | --- |
| Quest Diagnostics | 17813 | (TA)_n_ repeat | fPCR | <https://testdirectory.questdiagnostics.com/test/test-detail/17813/ugt1a1-gene-polymorphism-ta-repeat?p=r&q=UGT1A1&cc=MASTER> |
| Mayo Clinic | UGTFG | Full Gene | Sequencing | <https://www.mayocliniclabs.com/test-catalog/Overview/65428> |
| Mayo Clinic | U1A1V | (TA)_n_ repeat | fPCR | <https://www.mayocliniclabs.com/test-catalog/Overview/97400> |
| ARUP Laboratories | 3001755 | Full Gene | Sequencing | http://ltd.aruplab.com/Tests/Pub/3001755 |
| ARUP Laboratories | 51332 | (TA)_n_ repeat | Fragment Analysis | http://ltd.aruplab.com/Tests/Pub/0051332 |
| LabCorp | 511200 | (TA)_n_ repeat | PCR and Capillary Electrophoresis | <https://www.labcorp.com/test-menu/36281/iugt1a1-i-irinotecan-toxicity> |
| Washington University | 81350 | (TA)_n_ repeat | Fragment Analysis | https://pathology.wustl.edu/wp-content/uploads/2017/02/UGT1A1.pdf |
| University of Chicago | 81403 | Full Gene | Sequencing | <https://dnatesting.uchicago.edu/tests/crigler-najjar-syndrome-testing-ugt1a1> |
| University of Chicago | 81350 | (TA)_n_ repeat | PCR and sizing by capillary electrophoresis | https://dnatesting.uchicago.edu/sites/default/files/Gilbert%20Infosheet%206-2017.pdf |
| Baylor Genetics | 29535 | Full Gene | Next Gen Sequencing | https://www.bcm.edu/research/medical-genetics-labs/test_detail.cfm?testcode=29535&show=1 |
| Baylor Genetics | 29536 | (TA)_n_ repeat | Sequence Analysis | https://www.bcm.edu/research/medical-genetics-labs/test_detail.cfm?testcode=29536&show=1 |
| NIH Clinical Implementation (not currently testing UGT1A1) | N/A | N/A | DMET | https://www.ncbi.nlm.nih.gov/pubmed/28921647 |
| RPRD | Whole Pharmacogenomics Scan | (TA)_n_ repeat | Pharmacoscan or Next Gen Sequencing | https://www.rprdx.com/testing/overview/ https://www.rprdx.com/testing/whole-pharmacogenomics-scan-wps/#section7 |
| Qiagen | N/A | (TA)_n_ repeat | Therascreen UGT1A1 Pyrosequencing Kit | https://www.qiagen.com/punchout/detection-solutions/personalized-healthcare/therascreen-ugt1a1-pyro-kit/ |

**Table S1.** Available Testing Methods for Clinical Samples
