# Supplementary material for: Comparison of Eight Technologies to Determine Genotype at the UGT1A1 (TA)n Repeat Polymorphism: Potential Clinical Consequences of Genotyping Errors?
Source: Int J Mol Sci. 2020 Jan 30;21(3):896. doi: 10.3390/ijms21030896 (PMC7037496; doi:10.3390/ijms21030896)
Supplement: Supplementary file 1 [file ijms-21-00896-s001.zip › TABLE S2.docx]

**TABLE S1.** Percent and range of 5 fragment sizes determined by Illumina sequencing and fragment analysis

| (TA)*_n_*/(TA)*_n_* | %(TA)_4_ | %(TA)_5_ | %(TA)_6_ | %(TA)_7_ | %(TA)_8_ |
| --- | --- | --- | --- | --- | --- |
| *Illumina miSeq* |  |  |  |  |  |
| 5/6 (*n*=2) | 10.39 (9.59-11.20) | 71.24 (70.31-72.18) | 10.33 (10.15-10.51) | 0.26 (0.08-0.45) | 0 (N/A) |
| 5/7 (*n*=6) | 11.50 (9.32-13.26) | 77.63 (72.73-79.49) | 1.49 (1.05-2.42) | 1.60 (1.36-1.91) | 0.04 (0.008-0.07) |
| 6/6 (*n*=70) | 2.87 (1.95-5.27) | 20.21 (15.08-23.78) | 68.67 (56.33-72.90) | 0.86 (0.59-1.62) | 0.01 (0-0.05) |
| 6/7 (*n*=63) | 2.57 (1.22-4.19) | 17.67 (10.04-21.89) | 59.42 (33.34-64.70) | 9.58 (5.46-11.45) | 0.22 (0.11-0.37) |
| 6/8 (*n*=1) | 2.54 (N/A) | 18.94 (N/A) | 65.34 (N/A) | 2.70 (N/A) | 2.54 (N/A) |
| 7/7 (*n*=20) | 0.80 (0.30-1.14) | 5.58 (2.79-6.75) | 21.79 (13.28-23.97) | 59.28 (38.43-64.31) | 1.60 (1.08-1.83) |
| 7/8 (*n*=1) | 0.45 (N/A) | 4.73 (N/A) | 19.38 (N/A) | 52.73 (N/A) | 15.10 (N/A) |
| *Fragment Analysis* |  |  |  |  |  |
| 5/6 (*n*=7; *n*=3 unique) | 10.47 (9.08-11.71) | 78.56 (77.17-79.40) | 10.97 (10.05-13.75) | 0 (0) | 0 (0) |
| 5/7 (*n*=6) | 12.00 (10.41-13.16) | 84.33 (83.14-85.85) | 1.47 (0.99-1.67) | 2.20 (1.93-2.46) | 0 (0) |
| 6/6 (*n*=80; *n*=75 unique) | 1.99 (0-3.54) | 20.08 (18.38-23.06) | 77.10 (72.98-79.87) | 0.81 (0-1.55) | 0 (0) |
| 6/7 (*n*=69; *n*=64 unique) | 2.21 (0-5.10) | 17.83 (15.33-22.36) | 67.38 (61.70-70.58) | 12.57 (10.84-14.68) | 0 (0) |
| 6/8 (*n*=1) | 2.45 (N/A) | 18.45 (N/A) | 72.54 (N/A) | 2.67 (N/A) | 3.89 (N/A) |
| 7/7 (*n*=26; *n*=21 unique) | 0.49 (0-1.44) | 4.48 (3.77-5.45) | 21.36 (19.94-22.20) | 71.80 (67.80-74.51) | 1.87 (0-4.47) |
| 7/8 (*n*=1) | 0 (0) | 4.07 (N/A) | 16.29 (N/A) | 60.68 (N/A) | 18.96 (N/A) |
