# Supplementary figures and images for: Comparison of Eight Technologies to Determine Genotype at the UGT1A1 (TA)n Repeat Polymorphism: Potential Clinical Consequences of Genotyping Errors?
Source: Int J Mol Sci. 2020 Jan 30;21(3):896. doi: 10.3390/ijms21030896 (PMC7037496; doi:10.3390/ijms21030896)

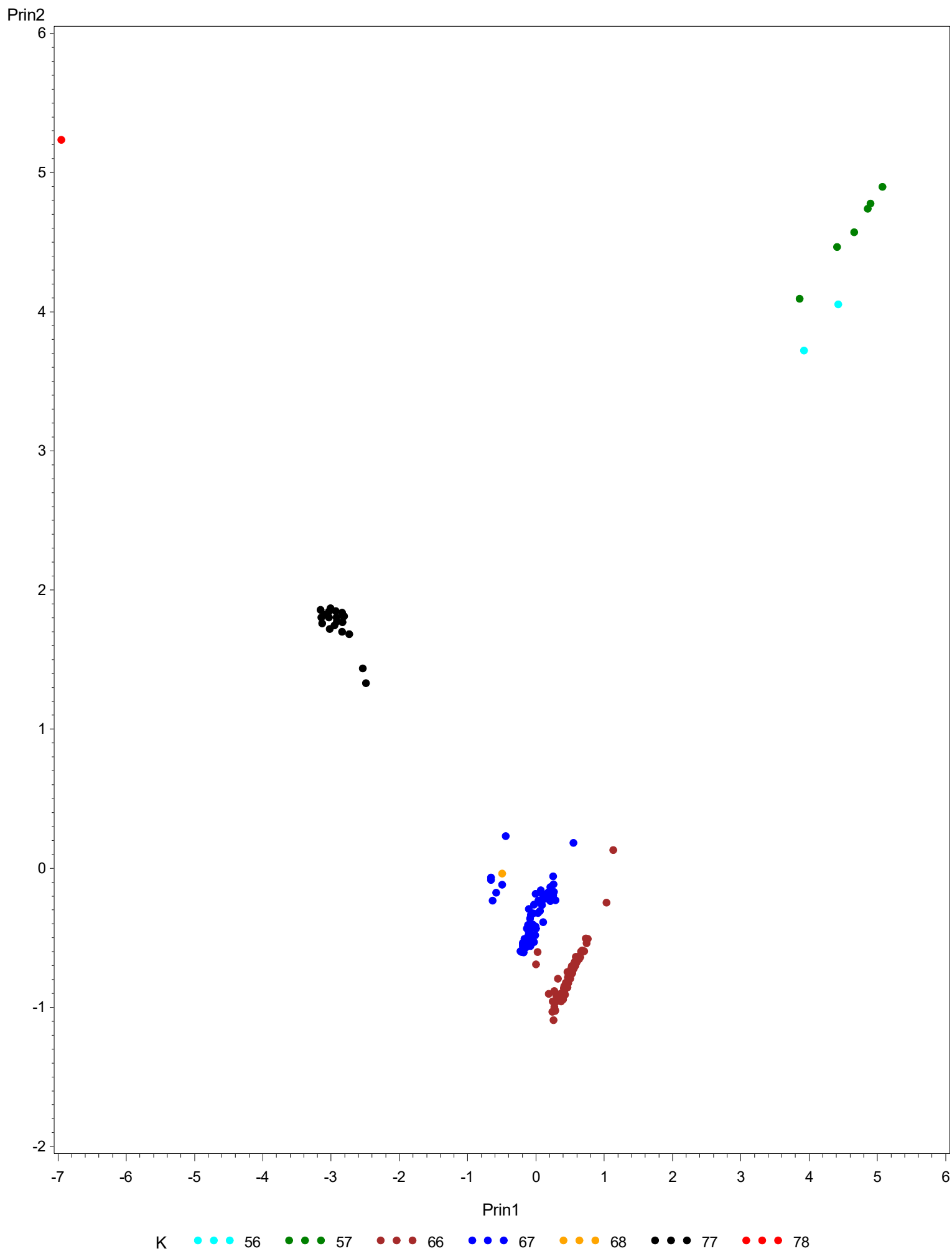

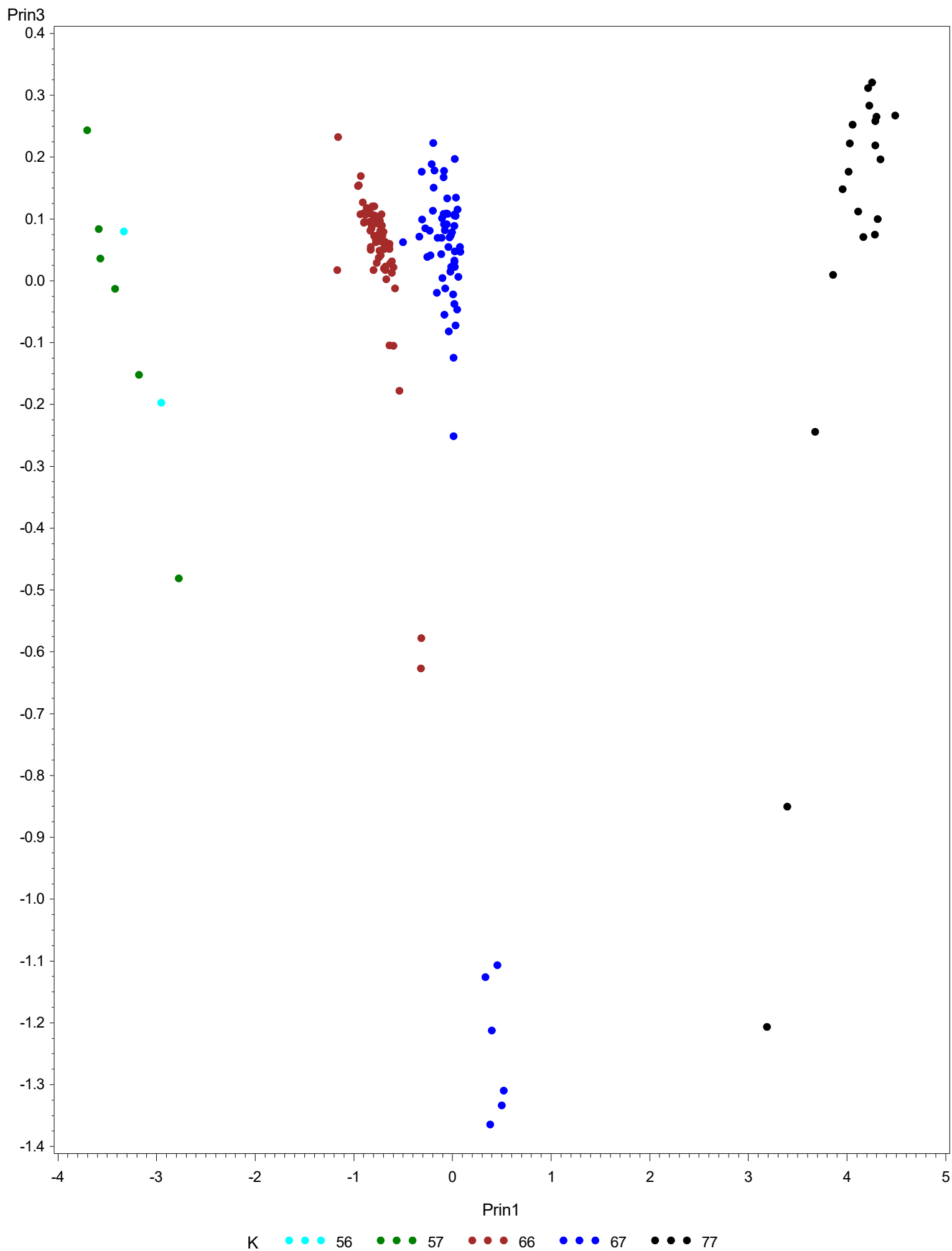

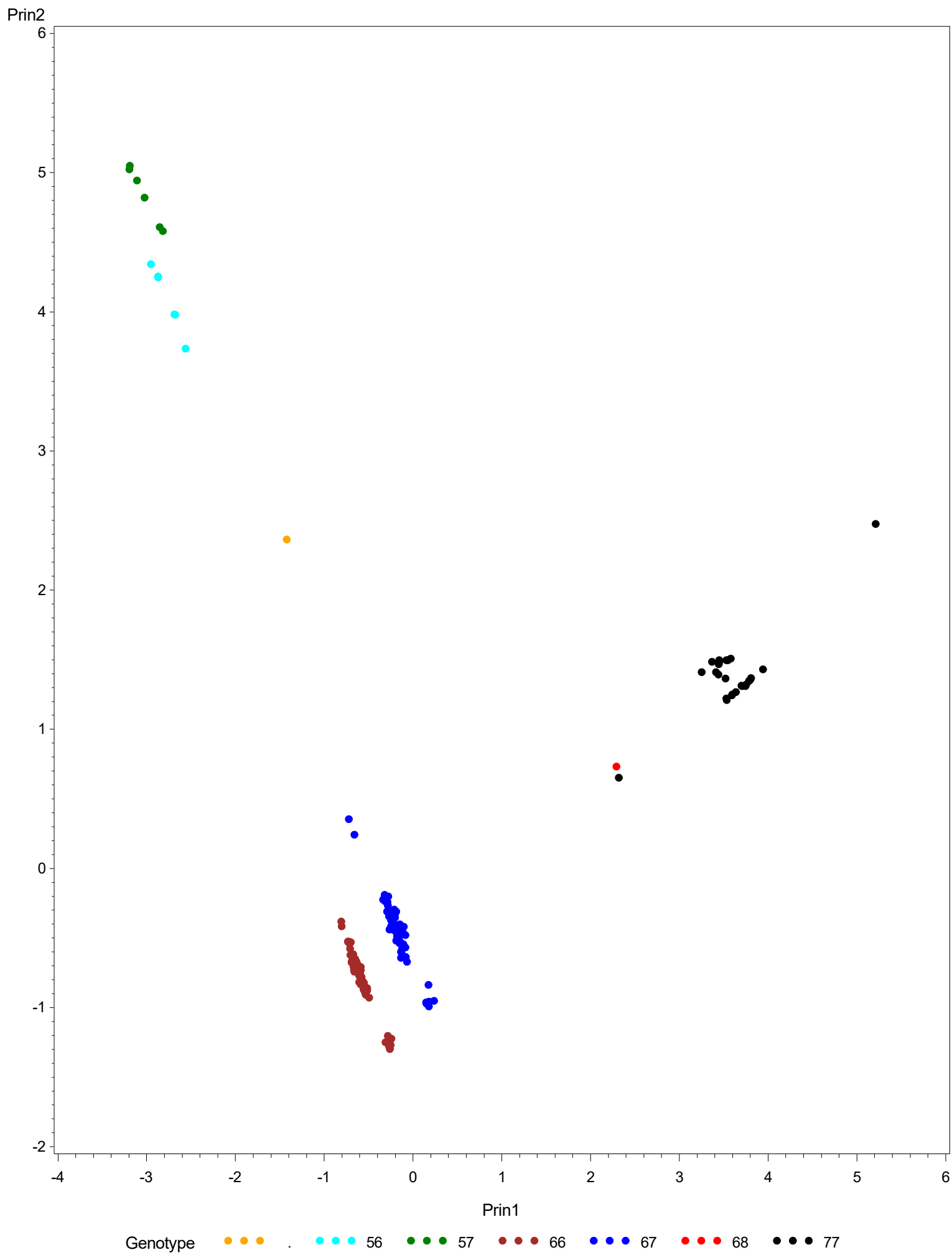

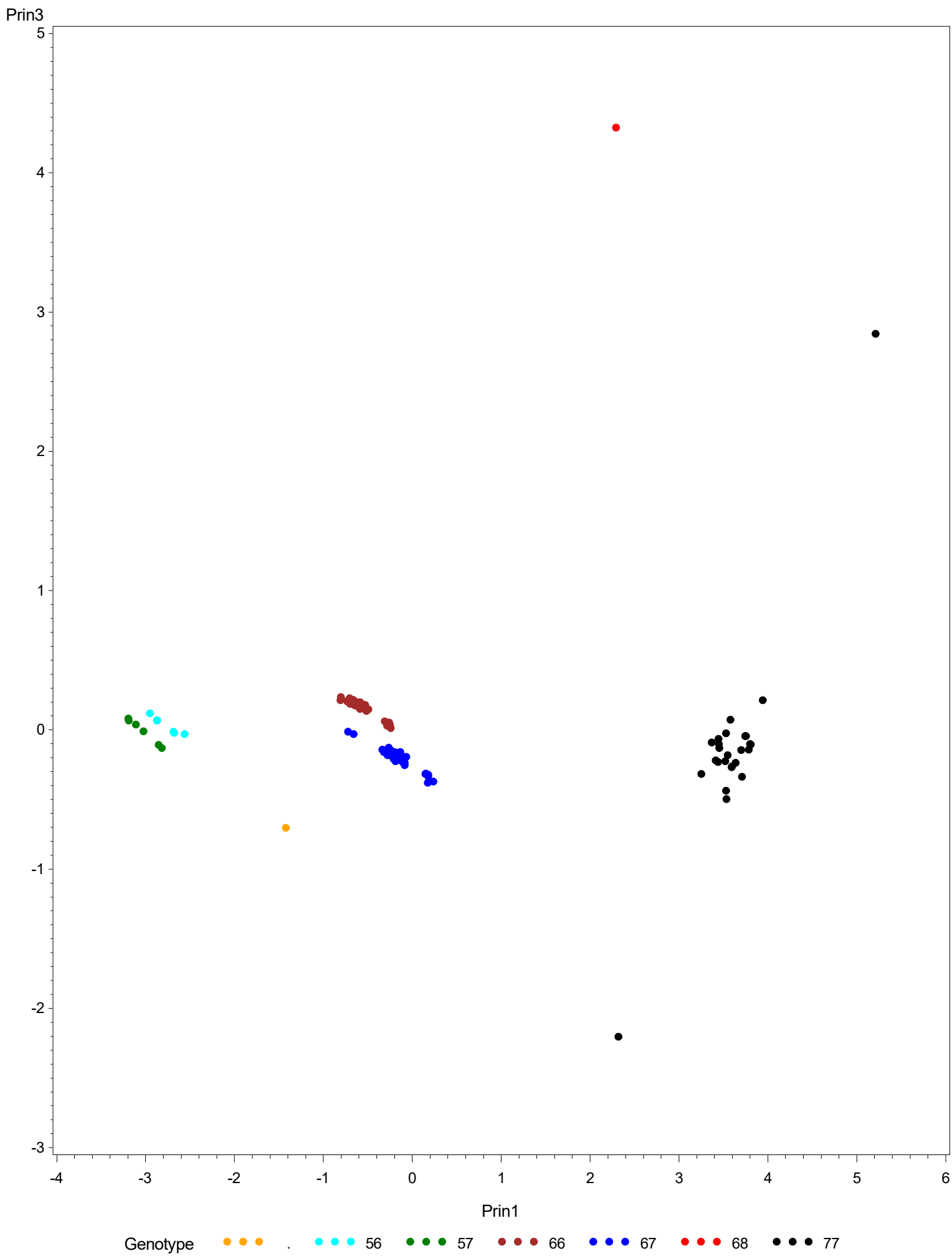

Supplement: Supplementary file 1 [file ijms-21-00896-s001.zip › Figure S1.pdf]
